# Metabolic host responses to malarial infection during the intraerythrocytic developmental cycle

**DOI:** 10.1186/s12918-016-0291-2

**Published:** 2016-08-08

**Authors:** Anders Wallqvist, Xin Fang, Shivendra G. Tewari, Ping Ye, Jaques Reifman

**Affiliations:** Department of Defense Biotechnology High Performance Computing Software Applications Institute, Telemedicine and Advanced Technology Research Center, U.S. Army Medical Research and Materiel Command, Ft. Detrick, MD 21702 USA

**Keywords:** Host-pathogen interactions, *Plasmodium falciparum*, Metabolism, Intraerythrocytic developmental cycle, Gene expression data, Oxidative stress response

## Abstract

**Background:**

The malarial parasite *Plasmodium falciparum* undergoes a complex life cycle, including an intraerythrocytic developmental cycle, during which it is metabolically dependent on the infected human red blood cell (RBC). To describe whole cell metabolic activity within both *P. falciparum* and RBCs during the asexual reproduction phase of the intraerythrocytic developmental cycle, we developed an integrated host-parasite metabolic modeling framework driven by time-dependent gene expression data.

**Results:**

We validated the model by reproducing the experimentally determined *1*) stage-specific production of biomass components and their precursors in the parasite and *2*) metabolite concentration changes in the medium of *P. falciparum*-infected RBC cultures. The model allowed us to explore time- and strain-dependent *P. falciparum* metabolism and hypothesize how host cell metabolism alters in response to malarial infection. Specifically, the metabolic analysis showed that uninfected RBCs that coexist with infected cells in the same culture decrease their production of 2,3-bisphosphoglycerate, an oxygen-carrying regulator, reducing the ability of hemoglobin in these cells to release oxygen. Furthermore, in response to parasite-induced oxidative stress, infected RBCs downgraded their glycolytic flux by using the pentose phosphate pathway and secreting ribulose-5-phosphate. This mechanism links individually observed experimental phenomena, such as glycolytic inhibition and ribulose-5-phosphate secretion, to the oxidative stress response.

**Conclusions:**

Although the metabolic model does not incorporate regulatory mechanisms *per se*, alterations in gene expression levels caused by regulatory mechanisms are manifested in the model as altered metabolic states. This provides the model the capability to capture complex multicellular host-pathogen metabolic interactions of the infected RBC culture. The system-level analysis revealed complex relationships such as how the parasite can reduce oxygen release in uninfected cells in the presence of infected RBCs as well as the role of different metabolic pathways involved in the oxidative stress response of infected RBCs.

**Electronic supplementary material:**

The online version of this article (doi:10.1186/s12918-016-0291-2) contains supplementary material, which is available to authorized users.

## Background

Despite extensive efforts to control malaria, the disease continues to kill >600,000 and sicken hundreds of million people annually [1]. Current strategies in vector control, advances in diagnostic techniques, and drug development have proven insufficient in controlling and eliminating malaria due to the emergence of resistance to existing drugs and the lack of effective prophylactic vaccines [1]. Fundamentally understanding the interactions of the malaria parasite with its hosts during its complex multistage life cycle have the potential to identify new key biological and physiological processes that could lead to new and improved antimalarial treatments. In the present study, we delineate strain-specific metabolism and address host-pathogen metabolic interactions that *Plasmodium falciparum*, the most virulent causative agent of malaria, engages in with its host environment [2].

The life cycle of *P. falciparum* includes a number of radically different host-dependent morphological stages [3]. It enters the human host through the bite of an infected *Anopheles* mosquito, where the infective sporozoites rapidly move to the liver and proliferate asymptomatically into merozoites. In turn, the merozoites invade red blood cells (RBCs) in the bloodstream, where some merozoites differentiate into sexual forms to reinfect mosquitos. However, the bulk of the merozoites undergo asexual reproduction during a ~48-h-long intraerythrocytic developmental cycle (IDC), which allows the parasites to infect many more RBCs. A tightly controlled development program characterizes the IDC, with one infecting merozoite undergoing 4–5 asexual reproduction cycles [4]. Initially, the merozoites establish themselves in a parasitophorous vesicle, shed invasion-specific organelles, and enter into the trophozoite form. The young trophozoite is initially termed a “ring”-stage, characterized by low metabolic activity, which after about 18 h post-infection rapidly grows and expands by consuming host metabolites to encompass the bulk of the infected erythrocyte volume. At around 30 h post-infection, the parasite enters the schizont stage, rapidly divides, and at ~48 h releases merozoites into the blood stream to complete the cycle.

During this process, *P. falciparum* alters key metabolic processes among infected and circulating RBCs to promote the colonization of the blood habitat. For example, *P. falciparum* inhibits two enzymes in the glycolysis pathway (phosphofructokinase [PFK] and pyruvate kinase [PYK]) in RBCs that are cocultured with infected RBCs but are not infected themselves, to decrease glucose utilization in the uninfected RBCs [5]. This inhibition ensures better glucose availability for the infected cells. Similarly, it could be reasonably assumed that the parasite also controls the glucose consumption of infected RBCs [6] to reserve this major energy source for itself. Presumably to compensate for the energy shortage in infected RBCs, *P. falciparum* also supplies ATP to its host through adenylate translocator proteins [7]. Furthermore, infected RBCs experience oxidative stress, which is intimately linked to glutathione metabolism and the pentose phosphate pathway [8]. These reactions and processes do not occur in isolation but, rather, are connected in a comprehensive systemic response. To unravel the metabolic components of the host response to malarial infection, we need a model that can simultaneously handle the coupled system of infected RBCs, uninfected RBCs, and the *P. falciparum* parasite itself. The technique of genome-scale metabolic network simulations provides the means to perform such system-level investigations.

Genome-scale metabolic networks are composed of interconnected biochemical reactions, each processing particular metabolites spontaneously or catalyzed through enzymes encoded by genes. Analyzing these networks under certain constrained conditions, such as limited nutrient uptake, allow for the prediction of cellular growth (biomass accumulation) and other phenotypic functions related to metabolism [9]. For example, metabolic networks for *P. falciparum* have been developed and used to identify essential genes/reactions that represent candidates for target-based antimalarial drug discovery [6, 10–14]. Importantly, these network descriptions have been used with IDC stage-specific gene expression data to instantiate ring-, trophozoite-, and schizont-specific metabolic networks to predict uptake or secretion of metabolites [12]. These studies were later extended by our group to capture stage-specific growth phenotypes and biomass metabolite production [15]. Among these metabolic descriptions, only the network model developed by Huthmacher et al. [6] includes explicit consideration of host metabolism within *P. falciparum*-infected RBCs. This model was designed to capture the stage-specific presence or absence of metabolic reactions in the network but does not address quantitative flux changes or the interplay between infected and uninfected RBCs. To overcome these limitations and to address the dynamic aspect of the host-pathogen metabolic interactions, we developed a model that predicts metabolic fluxes within both *P. falciparum* and its host RBCs at each hour during the IDC.

We created a new computational framework that explicitly takes into account genome-scale metabolic networks of both the parasite and the host RBC, metabolite exchange between these species, and uninfected RBCs to represent all components under *in vitro* culture conditions. Using the available experimental parasite gene expression data and media composition as the primary input, the model predicted metabolite utilization measured in the media during the IDC, including the major energy metabolites and amino acid uptake/secretion, to a high degree of accuracy. Energy metabolism, fluxes through the tricarboxylic acid cycle, and the accumulation of metabolite components into the biomass, i.e., the main constituent building blocks of the organisms, was strongly dependent on time and stage of the IDC, with high overall similarities between the *P. falciparum* strains HB3, 3D7, and Dd2, but also with noticeable differences in nicotinamide adenine dinucleotide (NAD), flavin adenine dinucleotide (FAD), protoheme, and polyamine processing, for example.

The addition of explicitly modeling host RBC metabolic reactions allowed us to explore how the infection of *P. falciparum* affected metabolism in both uninfected and infected RBCs. Because we do not capture all host-pathogen interactions that occur in infected RBC cultures, our approach was to effectively model known parasite actions, for example, by constraining specific metabolite fluxes, and capture the host metabolic consequences of these interactions. The direct manipulation of fluxes allowed us to predict the metabolic host alterations for otherwise unknown molecular or regulatory interaction mechanisms. These analyses suggested that the oxygen-releasing capability of uninfected RBCs cocultured with *P. falciparum*-infected RBCs was decreased, providing a molecular mechanism that could contribute to hypoxia in malarial infection. In addition, we used the model to describe the role of metabolism in the oxidative stress response of infected RBCs, which provided a rationale for why patients with enzyme deficiencies in the pentose phosphate pathway exhibit resistance to malaria.

## Methods

### Overview

**Fig. 1:**
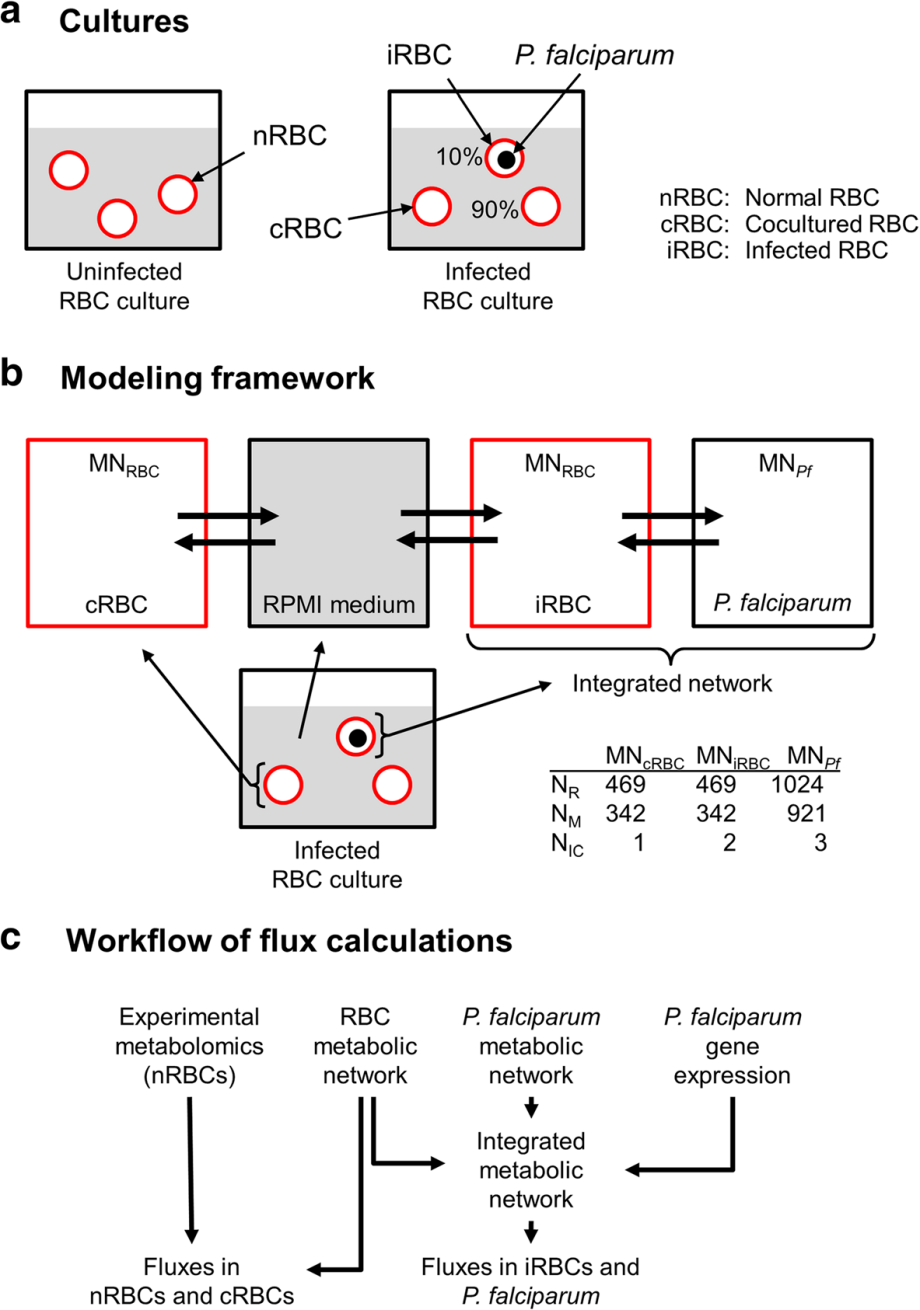
Schematic description for calculating metabolic fluxes in *Plasmodium falciparum* and human red blood cells. **a** Uninfected and infected human red blood cell (RBC) cultures. We simulated metabolic activity within RBCs for two cell culture conditions, i.e., an uninfected culture that consists of normal RBCs and an infected culture consisting of *P. falciparum*-infected RBCs and cocultured uninfected RBCs. **b** Modeling framework. In order to describe metabolism in the infected cultured system, we used separate metabolic network descriptions for each RBC component. The *P. falciparum* model was imbedded in a separate compartment of the infected RBC, allowing metabolite uptake and secretion between these entities. Direct metabolite uptake and secretion with the medium was only possible for the infected and cocultured RBC model. **c** Workflow of flux calculations. We used experimental metabolomic data of the uninfected RBC culture [16] to determine normal and cocultured RBC fluxes using the RBC metabolic network. As for infected RBCs, we combined RBC and *P. falciparum* metabolic networks into one integrated network and incorporated the parasite’s gene expression data to predict both host RBC and *P. falciparum* metabolic fluxes. cRBC, cocultured uninfected RBCs; iRBC, *P. falciparum*-infected RBCs; nRBC, normal RBCs; MN_*Pf*_, metabolic network of *P. falciparum*; MN_RBC_, metabolic network of RBC; N_IC_, number of internal compartments; N_M_, number of metabolites; N_R_, number of reactions; RPMI, Roswell Park Memorial Institute

We calculated metabolic fluxes in human RBCs for two different culture systems for which experimentally measured metabolomics data are available [16]. Figure 1a shows *1*) the uninfected culture, which consisted of normal RBCs in the absence of *P. falciparum*, and *2*) the infected culture, consisting of a mixture of *P. falciparum*-infected and uninfected RBCs. The infected culture condition was set to 10 % infected cells and 90 % uninfected cocultured RBCs [16].

Figure 1b shows the modeling framework and connections (metabolite secretion and uptake) between the components of the infected RBC culture. We used established metabolic networks with minor modification, as detailed below, to capture metabolic processes within cocultured and infected RBCs, where the *P. falciparum* metabolic network was embedded as a separate compartment in the infected cell. Exchange fluxes (thick arrows in Fig. 1b) account for uptake of nutrients and secretion of metabolites to the medium and between different compartments, including metabolite exchange between *P. falciparum* and the cytosol of the infected RBC.

Figure 1c shows the overall work flow of the flux calculations used to estimate metabolic activity within the different culture systems. We used experimental metabolomic data of the uninfected RBC culture [16] to determine fluxes in normal and cocultured RBCs based on the host RBC metabolic network (see Additional file 1: Text S1 and below). As one component of this work, we predicted fluxes within RBCs infected with each of three *P. falciparum* strains (HB3, 3D7, and Dd2). We estimated fluxes within both the host and parasite by combining the metabolic networks of the RBC [17] and *P. falciparum* [15] into one integrated network and, subsequently, for each strain, instantiating the network with the strain-specific expression data [18, 19]. Given the calculated fluxes in both cocultured and infected RBCs, we predicted the metabolite concentration changes for the infected RBC culture and validated the results using the available experimental data for the 3D7 strain [16]. Detailed descriptions of the data sets (including metabolic networks and gene expression data) and the computational procedures for flux estimations are given below.

The model framework explicitly accounts for metabolic and exchange reactions that are constitutively present in RBCs and the parasite, but leave out other mechanisms that mediate host-pathogen interactions. Although the detailed mechanisms behind these interactions are generally unknown, we can still use the developed framework to study the effect of these interactions. Thus, we have taken an effective approach where we constrain fluxes based on direct or indirect literature evidence and study the downstream effect of these perturbations in the context of the complete set of coupled metabolic reactions that constitute the computational model of the infected culture (Fig. 1b).

All scripts, codes, and data files used to generate the results are provided in http://bhsai.org/downloads/malaria/Additional file 2.zip.

### Metabolic networks

#### Host metabolic network

We started from the RBC metabolic network (*i*AB-RBC-283) developed by Bordbar et al. [17], which is a proteomic-based reconstruction that captures different metabolic pathways related to carbohydrates, nucleotides, amino acids, cofactors, and lipids. In addition to these pathways, we inserted a glutathione oxidization reaction to capture oxidative stress responses [20]. We also added reactions, including pantothenate kinase and Albumax II (a lipid-rich bovine serum albumin protein) degradation, to be able to capture and monitor the observed metabolite concentration changes in the medium (see Additional file 1: Text S1 for details). The final RBC model contained 469 reaction and 342 metabolites.

#### Parasite metabolic network

We used the parasite metabolic network model previously developed by us to describe the time-dependent metabolism of *P. falciparum* during the IDC [15]. This network, derived from the *i*TH366 network [12], includes a set of biomass functions instead of a single biomass objective function, allowing us to monitor the timing of the production of different biomass components [15]. We further modified this network by adding ATP secretion [7] and separating hemoglobin degradation into two reactions: the cleavage of hemoglobin into peptides and the degradation of peptides into free amino acids [21]. For the peptide degradation, we added the genes (PF14_0517, PF14_0439, PFI1570c, and MAL13P1.56) that encode the related aminopeptidase enzyme [22]. The final *P. falciparum* model contained 1024 reactions and tracked 921 metabolites in three different internal compartments: cytosol, mitochondria, and apicoplast.

#### The integrated host-parasite metabolic network

We developed an integrated malarial host-parasite network to represent the overall metabolism in *P. falciparum*-infected human RBCs. Formally, this network included all intracellular compartments in the *P. falciparum* metabolic network in a separate parasite compartment in the RBC. This construction allowed for metabolite exchanges between the parasite and host, as well as indirectly to the extracellular medium via the host cell (Fig. 1b).

We placed the intracellular reactions and metabolites of the *P. falciparum* network [15] into the corresponding cytosol, mitochondria, and apicoplast compartments of the parasite and replaced the original extracellular metabolites [15] with the corresponding materials from the RBC cytosol compartment. In addition, we added an ATP transport reaction from the parasite cytosol to the RBC compartment, allowing the secretion of this metabolite from the parasite into the host environment [7]. We assumed this secretion flux to be no more than 5 % of the ATP utilization rate by non-metabolic activities, setting the maximum secretion rate to 2.8 mmol/(h∙10^12^ RBC) [7].

We placed all intracellular reactions and metabolites of the original RBC network [17] into the RBC cytosol compartment. Based on the observation that the ratio of the reduced to oxidized glutathione levels in infected RBCs is roughly 10 times that of the ratio in normal RBCs [20], indicating a higher level of oxidative stresses faced by *P. falciparum*-infected RBCs, we forced the flux for the glutathione oxidization reaction to be 10 times its flux in normal RBCs. We also included transport reactions representing the metabolite exchanges between the RBC and extracellular compartments (medium) into the integrated network. This included all the original RBC transport reactions [17] as well as the transport reactions for *1*) the metabolites that exist in the extracellular compartment of the parasite network [15] but are missing in the host network [17] and *2*) metabolites whose uptake/secretion require ATP or other cofactors through membrane transport pathways induced by *P. falciparum* in the infected RBC [23].

#### Gene expression data for *P. falciparum* during the IDC

Similar to our previous approach [15], we used gene expression data collected hourly from synchronized populations of *P. falciparum* during the IDC [18, 19]. Here, we included data for all three *P. falciparum* strains (HB3, 3D7, and Dd2) to compare strain-specific metabolic activity. We processed the time-series gene expression data to account for the mRNA/protein mismatch using the experimentally measured time difference between when a gene was transcribed and when the synthesized proteins appeared [24] to shift the gene expression data to when the enzymes appear [15]. The outcome of this procedure was an expression level *r*_*j*_^*t*^ for each reaction *j* at each time point *t* that is representative of the corresponding enzyme activity.

#### Predicting metabolic fluxes in cocultured RBCs

We estimated metabolic fluxes (*v*_*j*_^*cRBC*^) in cocultured RBCs by solving for *v*_*j*_ in the following optimization problem:

1 $$ \min \kern0.5em {\displaystyle \sum_{j\in RBC}{\omega}_j\left|{v}_j-{v}_j^{nRBC}\right|} $$

$$ \mathrm{s}.\mathrm{t}.\kern0.5em {\mathbf{S}}_{RBC}\cdot \mathbf{v}=\mathbf{0} $$

$$ \mathbf{lb}\le \mathbf{v}\le \mathbf{u}\mathbf{b} $$

$$ {v}_j\le f\cdot {v}_j^{nRBC}\ j = \mathrm{P}\mathrm{Y}\mathrm{K}\ \mathrm{or}\ \mathrm{P}\mathrm{F}\mathrm{K} $$

where *v*_*j*_^*nRBC*^ represents the predicted fluxes in normal RBCs (see Additional file 1: Text S1 for the calculation of *v*_*j*_^*nRBC*^), *ω*_*j*_ is a coefficient for each reaction *j* of the objective function, **v** denotes the vector of reaction fluxes in the host metabolic network and its component *v*_*j*_ represents the flux through reaction *j* in units of mmol/(h∙10^12^ RBC), **lb** and **ub** indicate the lower and upper bounds of these fluxes, respectively, and **S**_*RBC*_ indicates the stoichiometric matrix for the host metabolic network. The special cases of PFK and PYK fluxes account for their inhibitory effect *f* on uninfected RBCs in the same culture [5].

We set *ω*_*j*_ to *1*) 0.0 for all transport reactions, *2*) 1.0 for non-glycolytic intracellular reactions, and *3*) 0.5 for glycolytic reactions based on the assumption that the RBCs have some leeway in adjusting the glycolytic function given that other intracellular functions are maintained. The sensitivity of the latter approximation was tested by systematically examining values of *ω*_*j*_ ranging from 0.1 to 0.9 and observing no material changes in the computational results. Finally, we set the value of *f* to 0.19 to match the relative glucose uptake rate observed for coinfected compared to normal RBCs (16 %) [5].

#### Predicting metabolic fluxes within infected RBCs

We defined a nested set of optimization problems to estimate metabolic fluxes of infected RBCs. Specifically, we calculated flux *v*_*j*_^*t*^ at each time point *t* for each reaction *j* for both the host and parasite by initially minimizing the difference *J*_*t*_ between the reaction fluxes and the product of their nominal fluxes and expression values as follows:

2 $$ \min \kern0.5em {J}_t={\displaystyle \sum_{j\in G}\left|{v}_j^t-{r}_j^t\cdot {v}_j^N\right|} $$

$$ \mathrm{s}.\mathrm{t}.\kern0.5em \mathbf{S}\cdot {\mathbf{v}}^t=\mathbf{0} $$

$$ \mathbf{lb}\le {\mathbf{v}}^t\le \mathbf{u}\mathbf{b} $$

$$ {v}_j\le f\cdot {v}_j^{nRBC}\ j = \mathrm{P}\mathrm{Y}\mathrm{K}\ \mathrm{or}\ \mathrm{P}\mathrm{F}\mathrm{K}\ \mathrm{in}\ \mathrm{RBCs} $$

where *v*_*j*_^*N*^ represents the nominal flux through reaction *j* in *P. falciparum* as defined and calculated in our previous work [15], *G* represents the set of the parasite’s intracellular irreversible reactions that can be associated with gene expression data, and **S** denotes the stoichiometric matrix for the host-parasite integrated network.

We further selected the solution that best maintained the host metabolic functions and was closest to the nominal flux distribution of the parasite by solving the following additional optimization problem:

3 $$ \min \kern0.5em {\displaystyle \sum_{j\in H}{\omega}_j\left|{v}_j-{v}_j^{nRBC}\right|}+{\displaystyle \sum_{j\in P}\left|{v}_j^t-{v}_j^N\right|} $$

$$ \mathrm{s}.\mathrm{t}.\kern0.5em \mathbf{S}\cdot {\mathbf{v}}^t=\mathbf{0} $$

$$ \mathbf{lb}\le {\mathbf{v}}^t\le \mathbf{u}\mathbf{b} $$

$$ {v}_j\le f\cdot {v}_j^{nRBC}\ j = \mathrm{P}\mathrm{Y}\mathrm{K}\ \mathrm{or}\ \mathrm{P}\mathrm{F}\mathrm{K}\ \mathrm{in}\ \mathrm{RBCs} $$

$$ {\displaystyle \sum_{j\in G}\left|{v}_j^t-{r}_j^t\cdot {v}_i^N\right|}\le {J}_t^{*} $$

where *H* and *P* in the objective function denote the host and parasite components of the integrated network, respectively, *J*_t_^*^ represents the optimal value for the objective function from the previous optimization problem defined by Eq. 2, and the last constraint ensures that this solution is one of the optimal solutions for Eq. 2.

Given the metabolic flux distributions for all time points obtained by solving Eqs. 1, 2 and 3, we finally determined the overall biomass production level *μ*^*t*^ of *P. falciparum* in human RBCs at each time point *t* as follows:

4 $$ {\mu}^t=\frac{{\displaystyle \sum_{j\in B}{v}_j^t{w}_j}}{{\displaystyle \sum_{j\in B}{w}_j}} $$

where *B* denotes the set of biomass functions [15] and *w*_*j*_ indicates the biomass fraction of the metabolite(s) associated with biomass function *j*. We defined *w*_*j*_ as follows:

5 $$ {w}_j={\displaystyle \sum_i\frac{c_{ij}{W}_i}{1000}} $$

where *c*_*ij*_ represents the coefficient of metabolite *i* in biomass function *j*, *W*_*i*_ denotes the molecular weight of the metabolite, and the factor 1000 converts moles into millimoles.

#### Comparison with experimental metabolomic data

We validated our model by comparing predicted time-dependent metabolite concentrations in the medium of *P. falciparum* 3D7-infected human RBC cultures with the corresponding experimental data. We obtained the experimental concentrations for 24 metabolites at 0, 8, 16, 24, 32, 40, and 48 h during the IDC by multiplying each metabolite’s experimental normalized concentrations (initial levels were normalized to one) in the culture medium at each time point [16] with their corresponding original concentrations in Roswell Park Memorial Institute (RPMI) 1640 medium [25].

We calculated concentrations *X*^*t*^_*i*,sim_ for metabolite *i* at the time points *t* = 0, 8, 16, 24, 32, 40, and 48 h from the following equation:

6 $$ {X}_{i,\mathrm{s}\mathrm{i}\mathrm{m}}^t={X}_i^0+{\displaystyle \sum_{\tau =1}^t\left(0.1{v}_{i,\mathrm{i}\mathrm{E}\mathrm{X}}^{\tau }+0.9{v}_{i,\mathrm{c}\mathrm{E}\mathrm{X}}^{\tau}\right)\cdot \Delta t\cdot \left[RBC\right]} $$

where *X*_*i*_^*0*^ represents the initial concentration in the medium (mmol/l) [25], *τ* indicates the hourly time points up to time *t*, *v*^*τ*^_*i*,iEX_ and *v*^*τ*^_*i*,cEX_ denote the exchange fluxes of metabolite *i* at time point *τ* for infected and cocultured RBCs in mmol/(h · 10^12^RBC), respectively, Δ*t* is the time interval of 1 h, and [*RBC*] represents the total RBC concentration in the culture, which was equal to 0.11 · 10^12^ RBCs/l based on a hematocrit of 1 % [16]. The coefficients 0.1 and 0.9 are the fractions of infected and cocultured RBCs among the whole RBC population, as defined by the 10 % parasitemia [16]. We obtained the values for *v*^*τ*^_*i*,cEX_ and *v*^*τ*^_*i*,iEX_ by solving Eqs. 1 and 3, respectively, and their positive and negative values indicate secretion and uptake fluxes, respectively.

## Results

By combining the metabolic network of the malarial parasite *P. falciparum* [15], the network of its host cells (human RBCs) [17], and the parasite’s gene expression data during the IDC [18, 19] (see Methods for details), we developed an integrated host-parasite metabolic model that explicitly accounted for the metabolic activity within both *P. falciparum* and RBCs at each hour during the IDC. We used the modeling framework shown in Fig. 1b to predict metabolic fluxes in RBC cultures infected with each of three *P. falciparum* strains (HB3, 3D7, and Dd2), including the fluxes for biomass production and metabolite uptake/secretion.

We organized the results into an initial set of model validation studies focused on comparison with strain-specific *P. falciparum* metabolic activity and RBC metabolism in infected cultures based on existing experimental data. We then turned to investigating host-pathogen interactions using the uninfected and infected RBC models to highlight altered metabolic activities associated with the effects of glycolytic inhibition and oxidative stress associated using the 3D7 strain as a model organism.

### Comparison and validation of *P. falciparum* metabolism

#### Comparison of standalone and integrated models of HB3 metabolism

We initially compared the results from the integrated RBC/parasite model with those derived from a simplified description of the system based on solely modeling *P. falciparum* HB3 metabolism, i.e., without the explicit host-pathogen coupling [15]. We calculated absolute differences in metabolic fluxes of the parasite and time-course correlation of fluxes across the IDC to assess whether the explicit host-pathogen coupling *per se* introduced any anomalies in parasite metabolism. The mean of the absolute differences between the two sets of fluxes was 5 % of the average absolute fluxes based on the parasite-only model, and the correlation between time-series fluxes had a mean and standard deviation of 0.88 and 0.27, respectively, suggesting that the explicit metabolic coupling pursued here did not quantitatively change internal *P. falciparum* metabolism *per se*.

#### Comparison of strain-specific metabolism

**Fig. 2:**
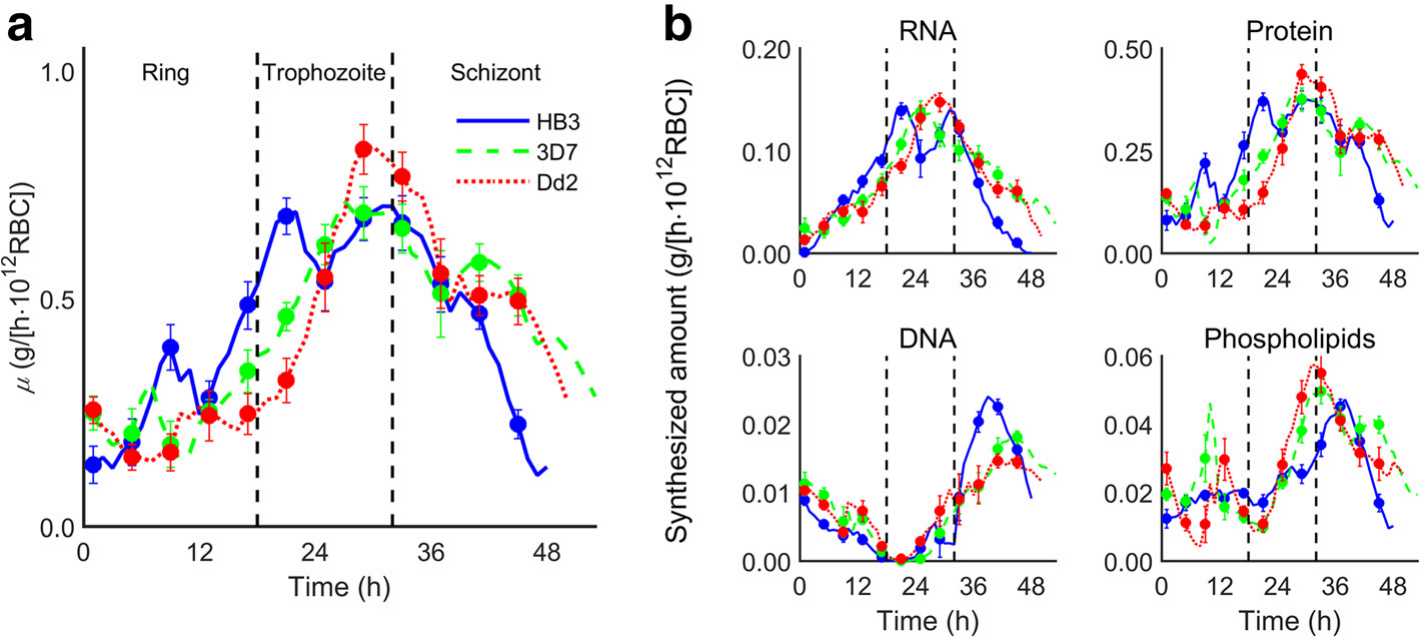
Predicted overall biomass production rates *μ* and macromolecular syntheses of *Plasmodium falciparum* during the intraerythrocytic developmental cycle. **a** Rates for the HB3 (*blue solid curve*), 3D7 (*green dashed curve*), and Dd2 (*red dotted curve*) strains of *P. falciparum* at each hour during the intraerythrocytic developmental cycle are shown. The whole intraerythrocytic developmental cycle was classified into ring, trophozoite, and schizont stages [19]. *μ* values are expressed as gram biomass per hour per 10^12^ red blood cells (g/[h · 10^12^ RBC]). **b** Synthesized amounts of RNA, protein, DNA, and phospholipids in the HB3, 3D7, and Dd2 strains of *P. falciparum* in g/(h · 10^12^ RBC). Error bars represent standard deviation (*N* = 20) of model uncertainty induced in response to 10 % Gaussian noise added to the gene expression data (Additional file 1: Text S2)

We examined overall biomass production rates for three different *P. falciparum* strains using the integrated RBC/pathogen model. Figure 2a shows biomass production rates *μ* of the HB3, 3D7, and Dd2 strains at each hour, encompassing all three IDC stages, i.e., ring, trophozoite, and schizont. The model predictions showed relatively low levels of biomass production rate for all strains during the ring stage, in agreement with the experimentally observed slow-growth phenotype associated with remodeling of internal structures during this stage [26, 27]. The predicted biomass production rates for all the strains were relatively higher during the trophozoite stage, during which the parasite grows rapidly through the consumption of the available nutrients in the infected RBC [28]. Finally, the schizont-stage biomass production rates decreased, compatible with the shifting focus of *P. falciparum* from cellular growth and accumulation of metabolites cell division [27].

We further compared macromolecular (RNA, protein, DNA, and phospholipids) synthesis during the IDC with *1*) the available experimental data for the HB3 strain [29, 30] and *2*) model results for the HB3, 3D7, and Dd2 strains. The comparison with the experimental data for the HB3 strain using the integrated model closely followed the agreement seen in the previously published data based on the standalone model (Additional file 1: Figure S1). Figure 2b shows the model results for strain-specific macromolecular syntheses during the IDC. We predicted that RNA and proteins were mainly synthesized by *P. falciparum* during the mid-IDC, whereas DNA and phospholipids were primarily produced during the late IDC, consistent with the corresponding experimental observations [29, 30]. The timing of the DNA and phospholipid syntheses corresponded to the production of genomic materials and cellular membranes required for cell division during the schizont stage.

To compare modeled metabolic activity among the three different strains, we calculated a pairwise Pearson correlation coefficient for each metabolite’s time-dependent flux, excluding reactions whose flux did not change during the intraerythrocytic development cycle (IDC). Table 1 shows significant correlations (mean *r* of 0.52–0.68 and *r*^*2*^ of 0.27–0.46) between time-series fluxes in each pair of strains. The high correlation is a reflection of the similarly highly conserved nature of the time-series gene expression data among strains, when extracellular and plasma membrane-associated genes were excluded [19]. Indeed, the mean between-strain correlation coefficient for the expression profiles of metabolic genes was 0.62–0.76 with an *r*^*2*^ of 0.38–0.58 (see Table 1). The overall decrease in flux correlations versus expression correlations is statistically significant and points to quantitative metabolic differences among the strains. Thus, despite the qualitative similarities that are expected based on the overall genomic strain similarities, we predicted the presence of quantitative strain difference at the metabolic level.

**Table 1:** Mean and standard deviation of Pearson correlation coefficients of reaction fluxes and metabolic gene expression data between different strains (HB3, 3D7, and Dd2) of *Plasmodium falciparum*

Comparison	Metabolic flux correlation coefficient		Metabolic gene expression correlation coefficient	
	Mean	Standard deviation	Mean	Standard deviation
HB3 vs. 3D7	0.55	0.24	0.62	0.26
HB3 vs. Dd2	0.52	0.24	0.72	0.25
3D7 vs. Dd2	0.68	0.23	0.76	0.26

A flux correlation coefficient was determined as the Pearson correlation coefficient between each strain pair’s time-series fluxes for each of the 530 reactions that were associated with non-constant fluxes. Similarly, we calculated gene expression correlation coefficients for the metabolic genes as the Pearson correlation coefficient between time-series expression data for each pair of strains. All pairwise strain differences between fluxes and gene expressions are statistically significant with *p*-values < 10^−4^

**Fig. 3:**
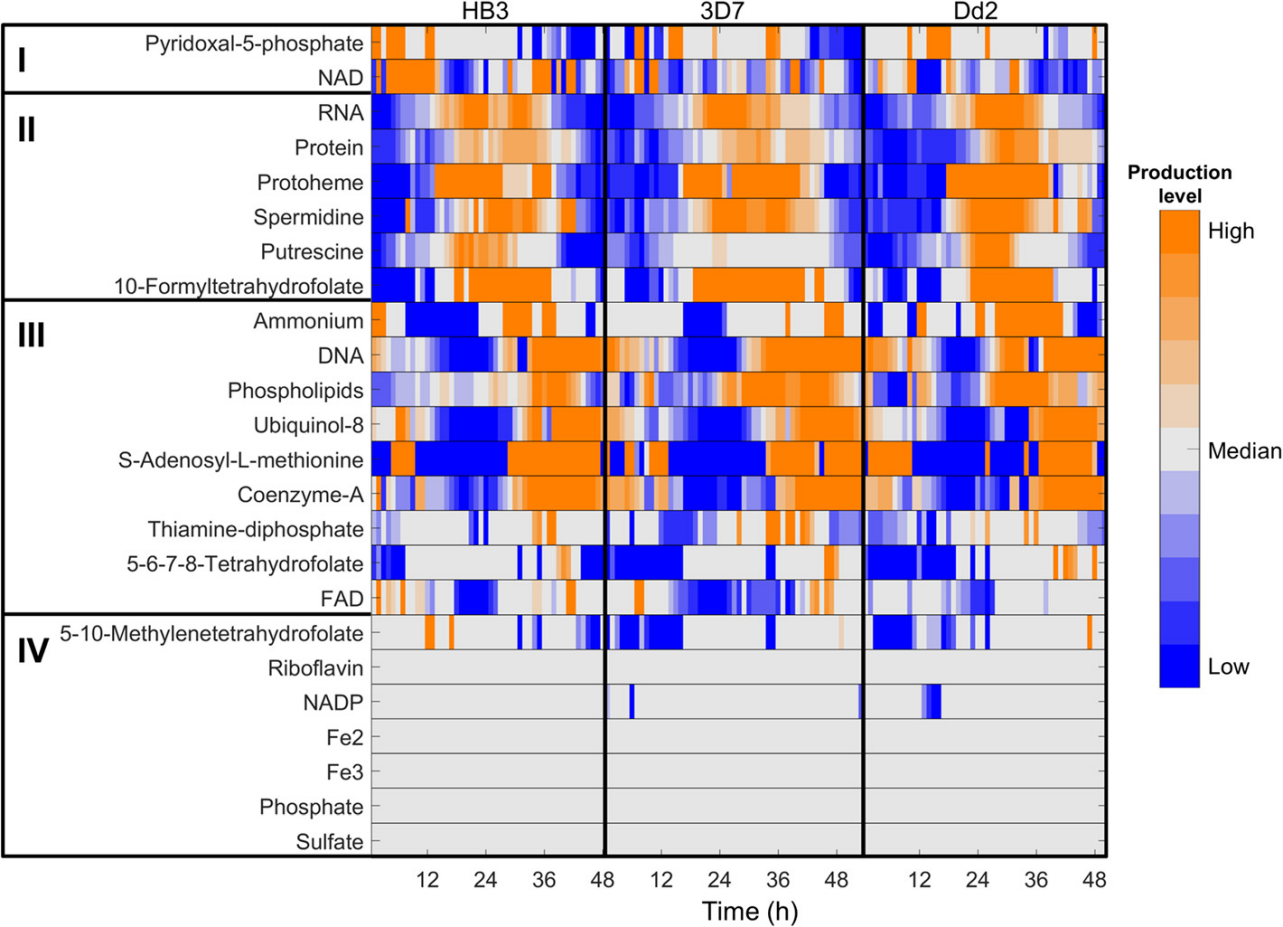
Predicted time-dependent production of biomass metabolites for the HB3, 3D7, and Dd2 strains of *Plasmodium falciparum*. The heat map denotes the predicted time-dependent production levels of each biomass metabolite of *P. falciparum*, in which orange, grey, and blue colors represent high, normal, and low production levels, respectively. Based on the time-dependent production, we classified these metabolites into four groups. *Groups I*, *II*, and *III* include the metabolites mainly produced during the early (ring stage), middle (trophozoite and early schizont stages), and late (schizont stage) periods of the intraerythrocytic developmental cycle, respectively, whereas *group IV* includes the metabolites for which the production levels were basically constant throughout the intraerythrocytic developmental cycle. Production value of each individual metabolite is normalized with respect to the median of its value for the HB3 strain

Figure 3 shows the time-dependent incorporation of metabolites into the biomass for the three strains studied. The detailed time courses for all biomass metabolites are shown in Additional file 1: Figure S2. As the strains execute similar transcriptional programs to process nutrients and prepare for cell division through the IDC via the metabolically distinct ring, trophozoite, and schizont stages, the data show both overall similarities and differences in stage-dependent metabolism. Given the variability of the expression data, consistently large and small differences appear among the strains, for example, in the NAD, FAD, protoheme, and polyamine processing between the strains. The HB3 strain was predicted to peak before leaving the ring stage compared to the more even production levels observed for the other strains throughout the IDC. Likewise, we predicted protoheme production levels to initiate and peak roughly 5 h earlier for the HB3 strain compared to 3D7 and Dd2. This indicated a potential differential strain-dependent sensitivity to drugs that target enzymes or pathways that produce these metabolites [31, 32]. Similarly, putrescine show different production rates indicating different metabolic strategies associated with polyamine accumulation before committing to schizogony: whereas 3D7 shows only a minor stage-dependent production rate variation, both HB3 and Dd2 show peak production rates in the late trophozoite stage. Although polyamine handing in *Falciparum* is not completely understood, the capacity to withstand polyamine depletion has been explored for anti-malarial drug development [33, 34]. Thus, the observed variability of putrescine production points to potentially large strain differences in effectively targeting polyamine-dependent processes.

**Fig. 4:**
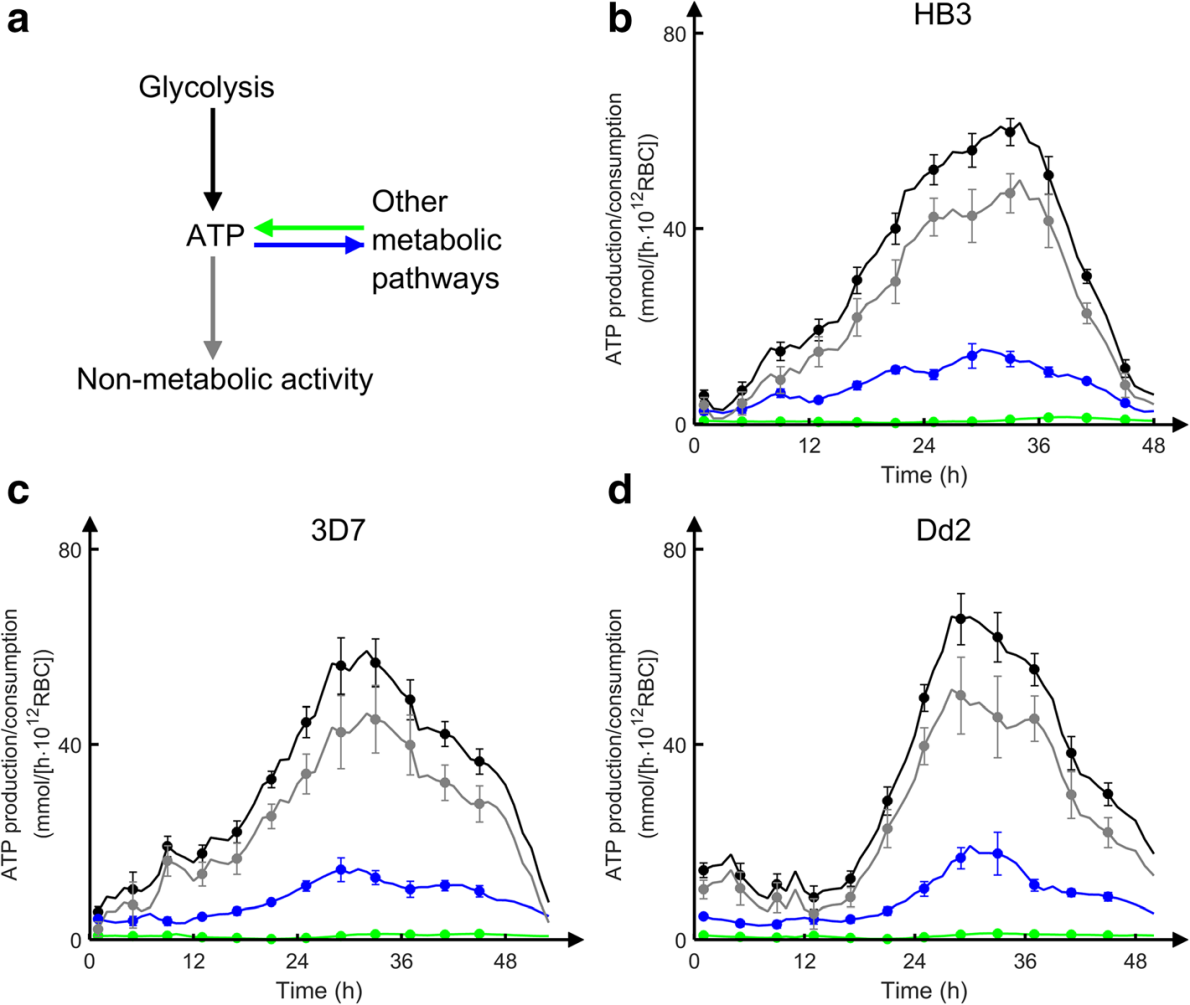
Predicted energy production and consumption in three strains of *Plasmodium falciparum*. **a** Schematic description of energy production and consumption. Energy (in the form of ATP) was produced from glycolysis (*black*) and other metabolic pathways (*green*) and consumed by non-glycolytic metabolism (*blue*) and non-metabolic activity (*grey*). **b**-**d**: Predicted time-dependent ATP production and consumption with respect to metabolic and non-metabolic processes (excluding ATP used for RNA synthesis) in the HB3 (**b**), 3D7 (**c**), and Dd2 (**d**) strains. Production or consumption are expressed as mmol/(h · 10^12^ RBC). Error bars represent standard deviation (*N* = 20) of model uncertainty induced in response to 10 % Gaussian noise added to the gene expression data (Additional file 1: Text S2)

Energy (in the form of ATP) was produced from glycolysis and other metabolic pathways and consumed by non-glycolytic metabolism and non-metabolic activity in a time- and strain-dependent manner. These processes exhibited a qualitative overall similarity, but with strain-specific differences. In particular, Fig. 4 shows differences in onset and peak ATP-production levels among the three strains. Whereas HB3 and 3D7 have an earlier onset of ATP production in the early trophozoite stage, Dd2 production was delayed but, once initiated, it rapidly compensated for the delayed onset by exhibiting the highest peak production level of 76 mmol/(h∙10^12^ RBC) at 28 h post infection. On the contrary, the reactions in the tricarboxylic acid (TCA) cycle all exhibited similar time-dependent flux profiles among the strains (see Additional file 1: Figure S3).

The modeling framework relies on gene expression data to drive parasite IDC metabolism using constrained stoichiometry defined by the *P. falciparum* metabolic network. The interpretation of the resultant metabolite changes rely on two main underlying assumptions, i.e., a correlation between mRNA and enzyme levels and limited non-transcriptional-based regulatory processes of metabolism. These assumptions are largely borne out by the observed general agreement with experimental data, but can also lead to non-biological model predictions, e.g., DNA production levels can continue into the ring stage from the schizont stage because of a lack of explicit cell-cycle regulation in the model. The correlation between mRNA levels and enzyme levels propagates high-frequency variations in mRNA levels to enzyme levels and, hence, variable metabolite levels. In reality, the transcriptional and translational machinery in the cell may not be able to respond quickly enough to these variations. We examined this latter point with respect to the biomass metabolites in more detail in Additional file 1: Text S2.

Although we did not identify any major qualitative metabolic differences during the IDC between the strains for the RBC culture condition examined here, the cross-species comparison showed potentially important strain differences. Thus, despite the expected high correlation among gene transcription between the strains, there was a significantly higher impact of these differences on the metabolic level.

### Experimental comparison of predicted metabolic activity in infected RBCs

**Fig. 5:**
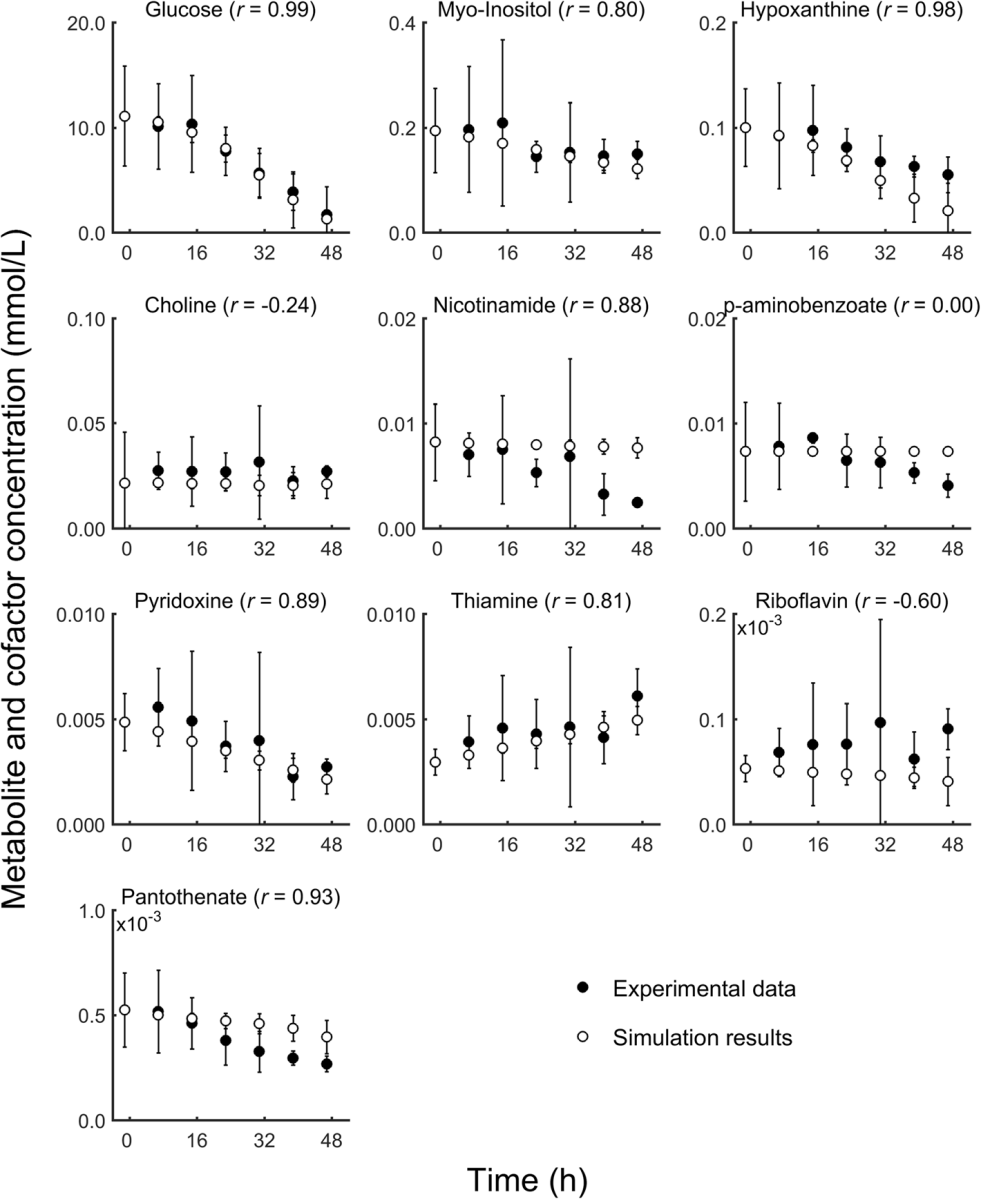
Extracellular metabolite concentrations for the *Plasmodium falciparum* 3D7-infected human red blood cell culture. Time-dependent computed (○) and experimental (●) concentrations of extracellular metabolites in the medium of the infected red blood cell (RBC) culture during the intraerythrocytic development cycle (IDC). Increasing values indicate secretion, whereas decreasing values indicate uptake. Note that the initial concentration values at t = 0 h are set to the experimental values and the model predicts the concentration changes for t ≠ 0 h. Error bars represent 95 % confidence interval calculated as ± 1.96 σ/√ N, where the standard deviation σ was determined from the data and N represent the number of replicates. We used *N* = 3 experimental biological replicates and *N* = 20 simulation results, which were derived by adding 10 % Gaussian noise to the gene expression data (Additional file 1: Text S2)

**Fig. 6:**
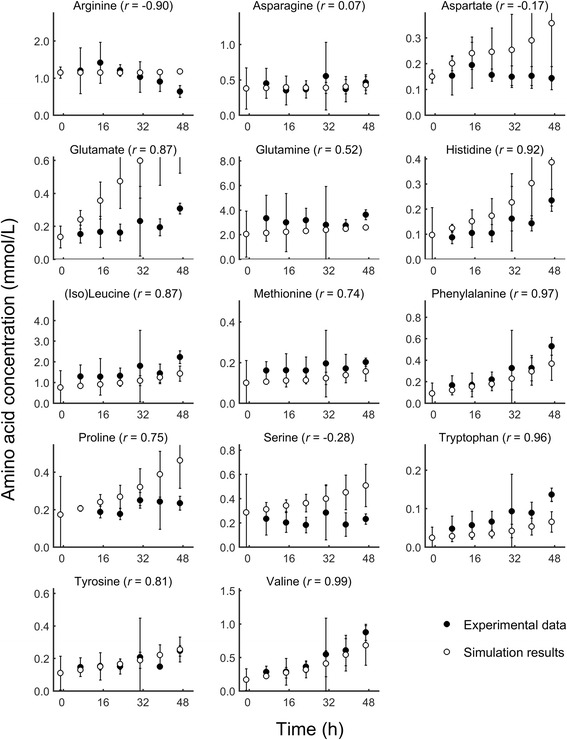
Extracellular amino acid concentrations for the *Plasmodium falciparum* 3D7-infected human red blood cell culture. Time-dependent computed (○) and experimental (●) concentrations of extracellular amino acids in the medium of the infected red blood cell (RBC) culture during the intraerythrocytic development cycle (IDC). Increasing values indicate secretion, whereas decreasing values indicate uptake. Note that the initial concentration values at t = 0 h are set to the experimental values and the model predicts the concentration changes for t ≠ 0 h. Error bars represent 95 % confidence interval calculated as ± 1.96 σ/√ N, where the standard deviation σ was determined from the data and N represents the number of replicates. We used *N* = 3 experimental biological replicates and *N* = 20 simulation results, which were derived by adding 10 % Gaussian noise to the gene expression data (Additional file 1: Text S2)

Compared with our previous work using a standalone model [15], the advantage of the new coupled host-pathogen model was that it allowed us to examine the metabolic activity of the infected host cell *per se*. To validate the modeled metabolic activity of the host cells, we compared time-course extracellular metabolite concentrations profiles predicted by our model to those derived from the experimentally measured extracellular metabolite data of a *P. falciparum* 3D7-infected RBC culture [16] (see Methods for details). This data set captures metabolite exchanges between the infected RBC and medium for 24 metabolites present in our model system. The metabolite concentrations in these experiments range in magnitude from 10^−4^ mM (pantothenate) to 10^1^ mM (glucose). Figure 5 shows the detailed time-course comparison for 10 major nutrients, cofactors, and nutrients sorted by concentration levels and Fig. 6 shows the time-course comparison for the measured 14 amino acids measured sorted alphabetically. Note that the concentration values at the initial time point t = 0 h were set to the experimental values and the model predicted the total concentration changes in the medium at subsequent times.

In the direct comparison between the measured and predicted concentration changes, the time-course behavior was captured for 16 out of the 24 metabolites with computed correlation values *r* exceeding 0.73, indicative of *p*-values < 0.05. For the metabolites and amino acids where the time-course behavior was not reproduced, the measured values tended to show less of a time dependency, partly obscured by the relatively large experimental uncertainty. These data reflect the cultured system and contain contributions from both the 90 % uninfected and 10 % *P. falciparum* infected RBCs.

To capture systemic metabolic differences due to the infection, we compared these results to a pure uninfected RBC culture as a control condition. Table 2 shows the initial and final metabolite concentration values in the medium for the control condition and the infected culture for the IDC, the difference between these values, and the qualitative contribution to secretion/uptake for the difference cultures. Note that in the control condition, there is no time variation in the rate of metabolite consumption.

**Table 2:** Medium metabolite and amino acid secretion and uptake comparison between uninfected control (Ctr) red blood cell (RBC) cultures versus *Plasmodium falciparum* (*Pf*) infected (Inf) RBC cultures

Metabolite	C _t=0 h_	ΔCtr_t=48 h_	ΔInf_t=48 h_	Δ*Pf* Inf	Secretion(+)/Uptake(−)	
	mM	mM	mM	mM	RBC	*Pf*
Glucose	1.11 10^+1^	−5.07 10^−1^	−9.97 10^+0^	−9.5 10^+0^	– – –	– – – –
Myo-Inositol	1.94 10^−1^	−7.29 10^−2^	−7.28 10^−2^	8.7 10^−5^	– –	– –
Hypoxanthine	1.00 10^−1^	−1.87 10^−2^	−8.09 10^−2^	−6.2 10^−2^	– –	– – –
Choline	2.15 10^−2^	4.05 10^−3^	−1.47 10^−3^	−5.5 10^−3^	+	–
Nicotinamide	8.19 10^−3^	0.00 10^+0^	−4.95 10^−4^	−5.0 10^−4^	○	–
p-aminobenzoate	7.29 10^−3^	0.00 10^+0^	0.00 10^+0^	0.0 10^+0^	○	○
Pyridoxine	4.86 10^−3^	−2.73 10^−3^	−2.73 10^−3^	0.0 10^+0^	–	–
Thiamine	2.96 10^−3^	2.18 10^−3^	1.99 10^−3^	−1.9 10^−4^	+	+
Riboflavin	5.31 10^−4^	0.00 10^+0^	−9.35 10^−5^	−9.3 10^−5^	○	–
Pantothenate	5.25 10^−4^	0.00 10^+0^	−1.25 10^−4^	−1.2 10^−4^	○	–
Amino acid	C _t=0 h_	ΔCtr_t=48 h_	ΔInf_t=48 h_	Δ*Pf* Inf	Secretion(+)/Uptake(−)	
	mM	mM	mM	mM	RBC	*Pf*
Arginine	1.148	0.012	0.026	1.5 10^−2^	+	+
Asparagine	0.378	0.074	0.036	−3.9 10^−2^	+	+
Aspartate	0.150	0.213	0.200	−1.2 10^−2^	++	++
Glutamate	0.136	0.848	0.726	−1.2 10^−1^	+++	+++
Glutamine	2.053	0.479	0.518	3.8 10^−2^	+++	+++
Histidine	0.097	0.090	0.279	1.9 10^−1^	+	++
(Iso)Leucine	0.762	0.324	0.647	3.2 10^−1^	+	+++
Methionine	0.101	0.021	0.052	3.1 10^−2^	+	+
Phenylalanine	0.091	0.143	0.268	1.2 10^−1^	+	++
Proline	0.174	0.149	0.280	1.3 10^−1^	+	++
Serine	0.285	0.149	0.210	6.2 10^−2^	+	++
Tryptophan	0.024	0.011	0.039	2.9 10^−2^	+	+
Tyrosine	0.110	0.106	0.140	3.4 10^−2^	+	++
Valine	0.171	0.191	0.491	3.0 10^−1^	++	++

The contribution to secretion (+) and uptake (−) from either RBCs or the parasite is qualitatively indicated in the last two columns by increasing number of +/− signs from low to high or none ○

C _t=0_, medium concentration at t = 0; ΔCtr_t=48 h_, change in concentration at t = 48 h for the uninfected control RBC culture; ΔInf_t=48 h_, change in concentration at t = 48 h for the *P. falciparum* infected RBC culture; Δ*Pf* Inf, ΔInf_t=48 h_ – ΔCtr_t=48 h_

The glucose concentration in the medium decreased as both RBCs and *P. falciparum* use it as their main source of energy during the ICD. Figure 5 shows the quantitative model predictions for this metabolite with a correlation coefficient *r* of 0.99 compared to the experimental data. Under the 48 h culture conditions studied here, uninfected RBCs consumed 0.5 mM of the initial amount at a constant rate whereas the infected culture consumed ~10 mM of the initial glucose at a stage-dependent rate. Thus, the observed large and time-dependent uptake of glucose uptake was mainly used to satisfy the energy requirement of the parasite, with the largest uptakes corresponding to the peak ATP demands shown in Fig. 4c.

The other two major metabolites taken up, myo-inositol and hypoxanthine, showed a general agreement with the experimental data. Myo-inositol uptake was largely driven by RBC metabolism, whereas hypoxanthine were taken up to larger degree in the infected cultures, consistent with the known hypoxanthine requirements for *in vitro* malaria growth [35]. The model predicted a small, but statically significant, increased hypoxanthine uptake at the later time points than what was observed experimentally. Hypoxanthine uptake is not tied to gene expression changes of any nucleoside transporter in the model, but is solely governed by the demands of the modeled metabolism. The uptake of hypoxanthine peaks in the trophozoite stage, commensurate with the peak in nucleic acid production and utilization, and provides the precursors of guanosine monophosphate that is required in nucleic acid synthesis. While sources for these precursors are available within the network model, the readily obtainable extracellular hypoxanthine provides the most parsimonious path to generate these intermediates.

Consistent with the experimental data there was a small choline secretion in the uninfected control condition [16], and a choline uptake in the infected culture consistent with the requirement for its uptakes by the parasite [36] (Table 2). The small variation of the overall concentration changes and the variability in the experimental data prevented a quantitative comparison (Fig. 5). We predicted nicotinamide uptake in the infected culture only, but not to the extent evident in the experimental data. We could not model the experimentally observed concentration changes of *p*-aminobenzoate because we did not find any independent information to support the addition of exchange reactions among the host, parasite, and medium for this metabolite to include in our model. The model predictions for pyridoxine uptake and thiamine (vitamin B1) secretion into the medium closely matched the experimental data, with the bulk of the pyridoxine uptake and thiamine secretion coming from the uninfected RBCs. In the infected culture, thiamine was secreted, but to a lesser extent than in the uninfected culture.

The model correctly predicted uptake of pantothenate, whereas the comparison of the predicted uptake of riboflavin (a required metabolite [12]) was obscured by experimental uncertainties for these data points. These metabolites were not taken up in the uninfected control culture.

Figure 6 and Table 2 show consistent model predictions for amino acid secretion during the IDC. The bulk of the secreted amino acids from the infected culture originated from the parasite and not from RBC metabolism (Table 2). The increased secretion was caused by the *P. falciparum* metabolic network component continuously synthesizing protein by metabolizing amino acids from the host hemoglobin, transporting the excess to the RBC, and, ultimately, to the medium [28]. The model displayed the largest lack of trend agreement for arginine, aspartate, and serine, i.e., the predicted increase in secretion was not present in the experimental data. To improve upon these predictions we will need additional metabolic information, in particular estimations of quantitative fluxes through arginase (for arginine) [16], asparagine synthase (for asparagine and aspartate) [12], and for currently unknown serine-related functions of the parasite metabolism to improve this aspect of the model.

In summary, the coupled host-pathogen metabolic network model showed a general good agreement with the available experimental data, and the bulk of the important metabolic processes and pathways showed quantitative agreements. Given the depth and sophistication of the model construct, we next examined how the infecting pathogen influences and manipulates host-cell metabolism in the cultured media.

### Host response to *P. falciparum*-induced glycolytic inhibition

Besides functioning as an exclusive energy source [37], the glycolysis pathway in human RBCs also includes a Rapoport-Luebering shunt, which generates and dephosphorylates 2,3-bisphosphoglycerate [38], a regulator of hemoglobin binding and release of oxygen [39]. Glycolysis within uninfected RBCs is affected by the presence of infected RBCs within the same medium [5]. In the presence of “conditioned medium,” i.e., the supernatant taken from a *P. falciparum*-infected RBC culture, uninfected RBCs have been observed to decrease their glucose consumption, lactate production, and activity of two glycolytic enzymes (PFK and PYK) [5]. To understand the metabolic implication of this effect, we incorporated the inhibition of PFK and PYK into our model, predicted the fluxes through the glycolytic enzymes of cocultured RBCs, i.e., uninfected RBCs cocultured with infected RBCs, and calculated the ratios of these fluxes to those of normal RBCs within a purely uninfected RBC culture.

**Fig. 7:**
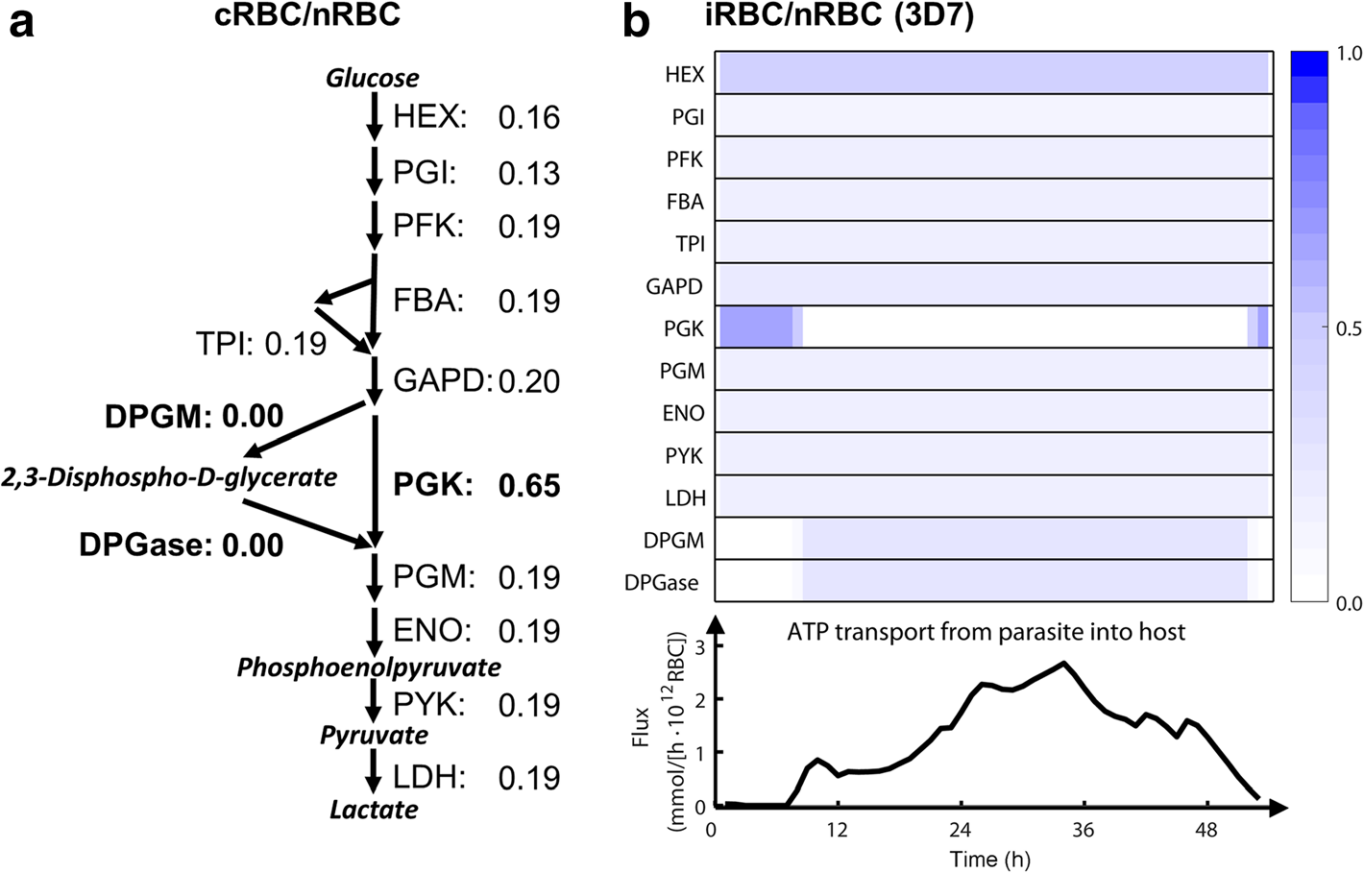
Flux ratios for reaction in the glycolysis pathways of human red blood cells. **a** The ratios of reaction fluxes in cocultured to those in normal red blood cells (RBCs). **b** Time-dependent ratios of reaction fluxes in infected RBCs to those in normal RBCs and time-dependent ATP transport from *Plasmodium falciparum* to its host RBC. ATP transport flux was expressed as mmol/(h∙10^12^ RBC). cRBCs, uninfected RBCs cocultured with iRBCs; DPGase, diphosphoglycerate phosphatase; DPGM, diphosphoglycero mutase; ENO, enolase; FBA, fructose bisphosphate aldolase; GAPD, glyceraldehyde-3-phosphate dehydrogenase; HEX, hexokinase; iRBCs, *P. falciparum* 3D7-infected RBCs in the infected RBC culture; LDH, lactate dehydrogenase; nRBCs, normal RBCs in the uninfected RBC culture; PFK, phosphofructokinase; PGI, glucose-6-phosphate isomerase; PGK, phosphoglycerate kinase; PGM, phosphoglycerate mutase; PYK, pyruvate kinase; TPI, triose-phosphate isomerase

Figure 7a shows that, for most of the glycolytic enzymes, the predicted fluxes in cocultured but uninfected cells were 13–19 % of those in normal cells, indicating a general inactivation of the glycolysis pathway induced by the presence of infected cells within the same culture. However, the ratio (0.65) for the phosphoglycerate kinase (PGK) enzyme was higher than for other enzymes, indicating resistance of the cocultured cells and an attempt to maintain this enzymatic function. Given that PGK converts one ADP molecule into one ATP molecule, this indicated that the ultimate purpose of this resistance might be to ensure an adequate energy supply for other biological processes in the RBC. In contrast to the relatively high PGK flux, we obtained zero ratios for diphosphoglycerate phosphatase (DPGase) and diphosphoglycero mutase (DPGM), the two enzymes in the Rapoport-Luebering shunt, suggesting significantly decreased fluxes through these enzymes or even a complete shutdown of the shunt. Given that DPGM and DPGase are responsible for the synthesis and decomposition of 2,3-bisphosphoglycerate, respectively, a RBC hemoglobin-oxygen binding regulator [39], our results implied a loss of oxygen-releasing capability of the cocultured RBC. In other words, we predicted that cocultured cells might maintain their energy support for survival by sacrificing their oxygen-releasing capability.

The predicted loss of oxygen-releasing capability of cocultured RBCs might be another mechanism that contributes to the hypoxic effects of malarial anemia. While one of the known mechanisms of malarial anemia is the sequestration by the spleen of infected and morphologically abnormal RBCs, it is still unclear of how the malarial parasite influences uninfected RBCs [40]. Other than the parasite-generated ligands and cytokines that increase the removal and decrease the production of uninfected RBCs, respectively [40], our results suggested a novel possible mechanism, i.e., *P. falciparum* might affect the glycolytic metabolism of uninfected RBCs by impairing their oxygen-releasing capabilities.

Importantly, the glycolytic pathways showed differential temporal activation among infected and cocultured RBCs. Figure 7b shows that, during the very early and late IDC, infected RBCs exhibited similar patterns to cocultured RBCs in terms of relatively high fluxes through PGK and low fluxes through DPGM and DPGase. However, during the middle IDC, we predicted low fluxes through the PGK enzyme in infected RBCs due to ATP secretion from *P. falciparum* [7] (Fig. 7b) when the parasite’s energy metabolism became active (Fig. 4). This might confer an advantage to the infected RBC, e.g., the added ATP [7] could partly compensate the inhibition of RBC glycolysis [5].

### Host response to *P. falciparum*-induced oxidative stress

Compared with un-infected RBCs, cells infected with *P. falciparum* confront higher levels of oxidative stress, caused by leakage of toxic free heme and reactive oxygen species from the hemoglobin degradation in the parasite as well as from oxygen radicals produced by the host immune response to malarial infection [41]. To gain insights into how infected RBCs respond metabolically to oxidative stress, we examined how our coupled host-pathogen model generated metabolic fluxes that alleviate the oxidative burden.

**Fig. 8:**
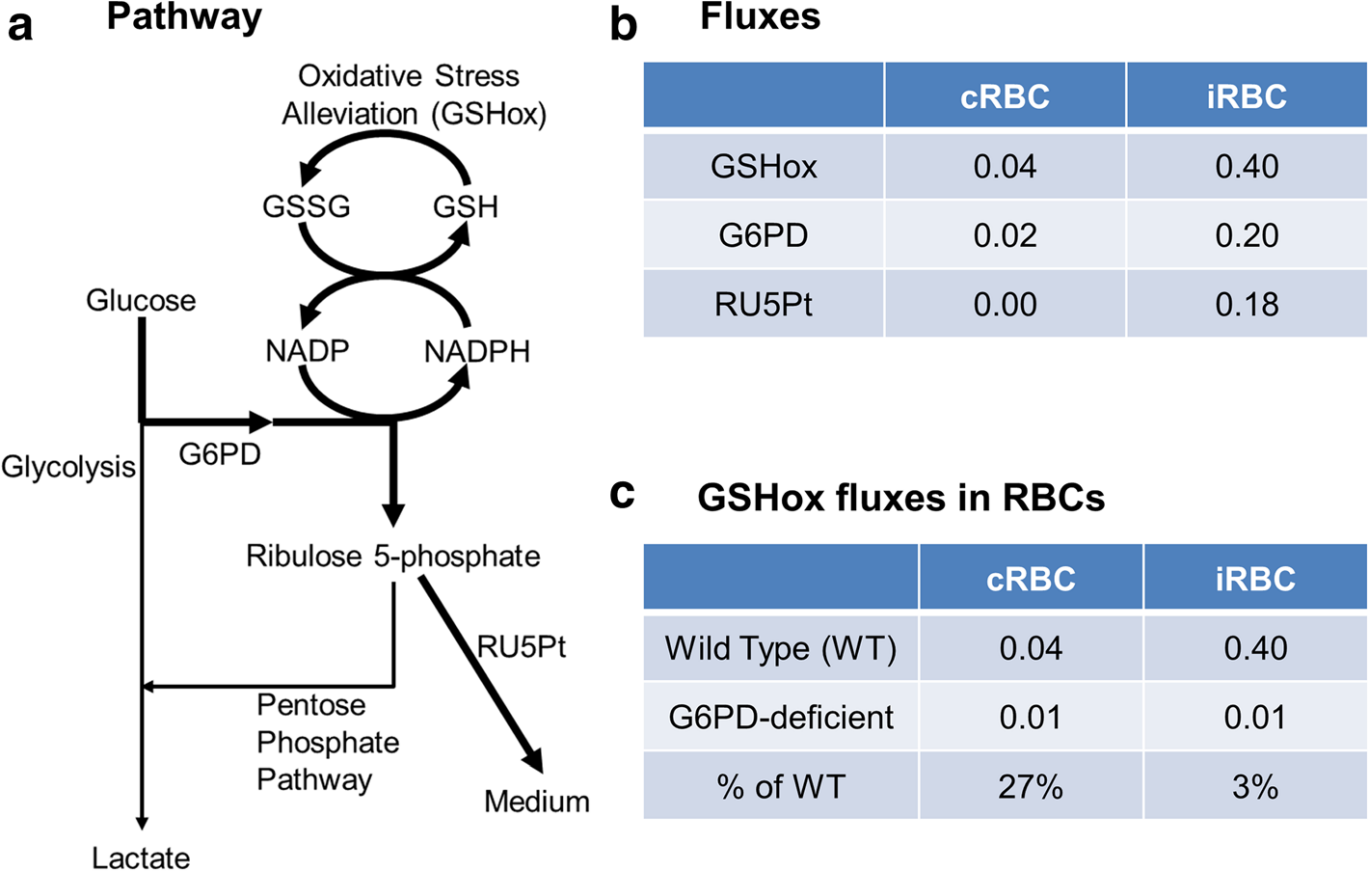
The pathway used for oxidative stress alleviation in *Plasmodium falciparum*-infected human red blood cells. **a** The pathway used by *P. falciparum*-infected red blood cells (RBCs) to deal with oxidative stresses. **b** Metabolic fluxes through the reactions of GSHox, G6PD, and RU5Pt within infected and cocultured RBCs, i.e., uninfected RBCs cocultured with infected RBCs. **c** GSHox fluxes within G6PD-sufficient (wild type) and G6PD-deficient RBCs. cRBCs, un-infected RBCs co-cultured with iRBCs; G6PD, glucose 6-phosphate dehydrogenase; GSH, reduced glutathione; GSHox, the GSH-based oxidative stress alleviation; GSSG, oxidized glutathione; iRBCs, *P. falciparum* 3D7-infected RBCs in the infected RBC culture; RU5Pt, the transport of ribulose 5-phosphate from iRBCs into the medium

Figure 8a shows the pathways used by infected RBCs in our model to handle the increased oxidative stress. These pathways used a series of reduction-oxidation reactions as well as part of the pentose phosphate pathway to ultimately reduce oxidative stress through the reduction of reactive oxygen reactive species (GSHox) by reduced glutathione (GSH). This process also simultaneously converted GSH to its oxidized form (GSSG), which, in turn, was reduced back to GSH through the oxidation of nicotinamide adenine dinucleotide phosphate (NADPH) to NADP by glutathione oxidoreductase. Finally, the reduction of NADP to NADPH was done by glucose 6-phosphate dehydrogenase (G6PD) and phosphogluconate dehydrogenase, two enzymes in the pentose phosphate pathway, which require an upstream flux from glucose and a downstream flux resulting in the secretion of ribulose 5-phosphate. Model predictions also pointed to important differences between cocultured and infected cells in the utilization of these pathways. For example, by using a higher GSHox flux in infected RBCs compared to cocultured RBCs, based on the observed ratio of reduced to oxidized glutathione levels in infected RBCs [20], we predicted that infected RBCs have higher fluxes through the G6PD reaction and secrets ribulose 5-phosphate (Fig. 8b).

The above pathway analysis implicated G6PD as an important enzyme related to oxidative stress in infected RBCs. Previous clinical observations have linked G6PD deficiency to resistance [42], but its underlying molecular mechanism is not clear. Here, we simulated G6PD deficiency by constraining the G6PD flux to be no greater than 26 % of that in normal RBCs [43] to understand its metabolic consequences. Figure 8c shows that G6PD-deficient cocultured RBCs were able to handle 27 % of their oxidative stress (represented by the flux through the GSHox reaction), whereas infected RBCs could only handle 3 %, indicating a relatively impaired survival capability of infected RBCs to survive within G6PD-deficient patients. This result is compatible with the observed resistance to malaria seen in G6PD-deficient patients [42], i.e., lack of G6PD impairs the ability of infected RBCs to survive oxidative stress, leading to RBC lysis and loss of a suitable host habitat for the parasite during the blood stage of malaria [44].

#### Connecting oxidative stress and metabolism

The coupled host-pathogen model simulation provided a comprehensive metabolic description of how infected RBCs respond to the parasite-generated oxidative stress. Given the increase in stress, infected host cells inhibited PFK and PYK (see Fig. 7a), two enzymes in the glycolysis pathway [5], in order to increase the flux from glucose through the host oxidative-phase pentose phosphate pathway. The ultimate product of this pathway was ribulose 5-phosphate, which, in turn, was secreted to the medium [16]. The requirement of the G6PD enzyme in this pathway provided a rationale for explaining why G6PD-deficient infected cells are less able to survive oxidative stress and, hence, why G6PD-deficient patients are more resistant to malaria [42]. Although it has been proposed that infected RBCs use the pentose phosphate pathway to handle oxidative stress [8], to date, no mechanistic links have been reported that link oxidative stress to glycolytic inhibition [5] and ribulose-5-phosphate secretion [41]. Thus, the coupled host-pathogen model allowed us to connect previous observations on *1*) the inhibition of the host glycolysis by the malaria parasite [5], *2*) the parasite-exacerbated oxidative stress faced by RBCs [41], *3*) the ribulose-5-phosphate secretion induced by *P. falciparum* infection [16], and *4*) the malarial resistance for G6PD-deficient patients [42] into a single coherent metabolic description of the oxidative stress response.

## Discussion

The life cycle of *P. falciparum* is complex, with two different hosts and multiple stages within each host. During the human blood stage, the asexual reproductive phase during the IDC takes place in infected RBCs, resulting in the familiar malarial symptoms of the disease. A key aspect of the host-pathogen interaction during the IDC is the heavy nutritional requirements faced by the rapidly dividing parasite. The host cell provides the environment and most nutrients necessary for the metabolism of the parasite, while at the same time, the parasite alters the metabolic activity of the host cell [5, 7, 8]. Previous *P. falciparum* metabolic network models have not captured these host-parasite metabolic interactions, although these models have correctly described the metabolism and growth phenotypes of the parasite during the IDC [6, 12, 15]. Thus, our effort focused on developing an integrated host-parasite metabolic model that allowed us to separately assess parasite and host metabolism in a culture consisting of infected and uninfected RBCs. We constructed the model by integrating the metabolic networks of both *P. falciparum* and human RBCs and using time course-dependent gene expression of the parasite during the IDC to drive the modeled metabolism.

### Parasite metabolism

Given the hourly gene expression data for three strains (HB3, 3D7, and Dd2) of *P. falciparum* [18, 19], we calculated metabolic fluxes in each of these strains at each hour during the IDC. These fluxes generally exhibited similar stage-specific time-series profiles for key metabolic processes, indicating no major qualitative differences among the strains under “normal” infected RBC culture conditions. This similarity among the different strains was largely due to their overall similar gene expression profiles. In spite of the highly correlated gene expression data, metabolic fluxes were significantly less correlated (*r*^*2*^ reduced by 20–30 %), indicating a quantitative less consistency in exactly how the metabolic program was executed during the IDC. We noted consistent strain-dependent differences among certain metabolites, e.g., NAD, FAD, protoheme, and polyamines, whereas others, such as 10-formyltetrahydrofolate, coenzyme-A, and thiamine-diphosphate, do not show strain-dependent differences. Differences in onset and peak ATP-production levels also differed noticeably among the three strains. Because the model provides the theoretical framework for describing metabolism, it requires condition-specific gene expression data to instantiate a particular condition. Thus, strain differences might become more important under different physiological stress or drug treatment conditions [19, 45].

### Host-pathogen interactions

The integrated host-pathogen metabolic model included the ability to describe separate populations of infected and uninfected RBCs in the same culture, allowing us to investigate the detailed and complex metabolic responses of the host. However, the underlying mechanisms by which the parasite regulates and affects cellular host processes, including both metabolic and non-metabolic are not always known. Here, we have taken an approach to directly implement such host-pathogen interactions by explicitly manipulating particular fluxes and modeling the downstream metabolic effects in our modeling framework.

Given the established glycolytic inhibition of uninfected RBCs under malarial infection [5], we predicted that uninfected RBCs strive to maintain their energy supply by decreasing the production of 2,3-bisphosphoglycerate, which is used by RBCs to regulate the oxygen binding of hemoglobin [39]. Therefore, the predicted decrease in the production of 2,3-bisphosphoglycerate indicated that the infection impaired the oxygen-releasing capability of uninfected RBCs, which could be a contributing factor to the observed hypoxic effects of malarial infections.

In addition, the inclusion of separate metabolic descriptions of infected and uninfected RBCs in our model allowed us to derive a comprehensive picture of how infected RBCs overcome the oxidative stress induced by the infection. We predicted that *P. falciparum* used the glycolysis pathway of infected RBCs to drive the metabolic flux from glucose to the pentose phosphate pathway. This process reduced parasite-induced reactive oxygen species and generated ribulose-5-phosphate as the final metabolic product, which was ultimately secreted to the environment. These predictions highlighted the importance of the G6PD enzyme in this process and provided a rationale for the observed malarial resistance of G6PD-deficient patients. To our knowledge, this is first description that links the oxidative stress response to a number of individual observations on parasite-induced metabolic changes in RBCs (the glycolytic inhibition [5], the oxidative stress increase [41], and ribulose-5-phosphate secretion [16]) as well as to the resistance of G6PD-deficient patients to malaria [42].

## Conclusion

During its life cycle, the malaria parasite sequentially progresses through multiple stages in female *Anopheles* mosquitos and humans. In humans, the blood stage of the disease is associated with the debilitating clinical symptoms of malaria. These symptoms are linked to the parasite undergoing synchronized asexual reproduction in RBCs, during which one parent cell multiplies into 16–32 daughter cells in ~48 h. Here, we developed a genome-scale, gene-expression-driven integrated host-pathogen metabolic network model that can capture and describe systemic changes in metabolism of the replicating malaria parasite *P. falciparum* as well as for infected and cocultured uninfected human RBCs. Although the modeling framework does not explicitly incorporate regulatory mechanisms, changes in the tightly controlled gene expression program of the parasite are sufficient to drive metabolic alterations in the modeling framework. This allowed us to predict not only strain-specific metabolic programs of *P. falciparum* during the reproductive cycle but also to examine parasite modulation of host metabolism in surrounding RBCs. Our system-level analysis suggested a primary metabolic similarity between the three studied strains, but also pointed to specific difference in metabolite production levels. Furthermore, our analysis revealed complex relationships such as how the parasite reduces oxygen-releasing capability of uninfected cells in the presence of infected RBCs as well as the role of different metabolic pathways involved in the oxidative stress response of infected RBCs.

## Abbreviations

cRBC, cocultured uninfected RBCs; DPGase, diphosphoglycerate phosphatase; DPGM, diphosphoglycero mutase; ENO, enolase; FAD, flavin adenine dinucleotide; FBA, fructose bisphosphate aldolase; G6PD, glucose 6-phosphate dehydrogenase; GAPD, glyceraldehyde-3-phosphate dehydrogenase; GSH, reduced glutathione; GSHox, the GSH-based oxidative stress alleviation; GSSG, oxidized glutathione; HEX, hexokinase; IDC, intraerythrocytic developmental cycle; iRBC, *P. falciparum*-infected RBCs; LDH, lactate dehydrogenase; MN_*Pf*_, metabolic network of *P. falciparum*; MN_RBC_, metabolic network of RBC; NAD, nicotinamide adenine dinucleotide; NADPH, nicotinamide adenine dinucleotide phosphate; N_IC_, number of internal compartments; N_M_, number of metabolites; N_R_, number of reactions; nRBC, normal RBCs; PFK, phosphofructokinase; PGI, glucose-6-phosphate isomerase; PGK, phosphoglycerate kinase; PGM, phosphoglycerate mutase; PYK, pyruvate kinase; RBC, red blood cell; RPMI, Roswell Park Memorial Institute; RU5Pt, the transport of ribulose 5-phosphate from iRBCs into the medium; TPI, triose-phosphate isomerase

## Additional file

Additional file 1: Supplementary computational details **Text S1-S2** and additional **Figures S1–S4**. (PDF 1107 kb)
