# Supplementary material for: Metabolic host responses to malarial infection during the intraerythrocytic developmental cycle
Source: BMC Syst Biol. 2016 Aug 8;10:58. doi: 10.1186/s12918-016-0291-2 (PMC4977726; doi:10.1186/s12918-016-0291-2)
Supplement: Additional file 1: — Supplementary computational details Text S1-S2 and additional Figures S1–S4. (PDF 1107 kb) [file 12918_2016_291_MOESM1_ESM.pdf]

Supplemental Materials

Table of Contents

Text S1. Calculation of metabolic fluxes in uninfected human red blood cells....................... 2

Text S2. Model sensitivity to high-frequency gene expression variations 10

Figure S1. Macromolecule syntheses in *Plasmodium falciparum* HB3 during the intraerythrocytic developmental cycle.. 12

Figure S3. Predicted metabolic fluxes through the tricarboxylic acid cycle in three strains of *Plasmodium falciparum* 14

Figure S4. Flux ratios for the reactions in the glycolysis pathways of human red blood cells infected with *Plasmodium falciparum* 3D7 and Dd2 strains. 15

References.. 16

Text S1. Calculation of metabolic fluxes in uninfected human red blood cells

Our objective was to obtained metabolic fluxes in human red blood cells (RBCs) that were neither infected nor cocultured with the malaria parasite *Plasmodium falciparum*. We did this by calculating a set of fluxes that satisfied the constraints in a human erythrocyte metabolic network [1] and had the best fit to increasing/decreasing rates of extracellular metabolite concentrations, as estimated from experimental metabolomics data of uninfected RBC cultures [2].

METHODS

We calculated the fluxes of uninfected RBCs through the following three steps.

***Step I**: Estimating experimental rates of extracellular concentration changes*

We first estimated the experimental means and 95% confidence intervals (CIs) of the relative change rates (in h^{-1}) of the extracellular concentrations for each of the 51 metabolites that were present in the metabolic network [1] and whose extracellular metabolomic data were experimentally determined [2]. The data included the means and standard deviations derived from triplicate measurements of each metabolite's extracellular concentration at 8, 16, 24, 32, 40, and 48 h relative to the level at time $t = 0$ [2].

For each metabolite, we calculated the mean and 95% CI of its relative change rate generated by 10,000 random simulations. For each simulation, we sampled the metabolite's relative concentration at each time point from a normal distribution with the corresponding mean and standard deviation [2] and performed a linear regression of the sampled time-series concentrations to determine the relative change rate.

We further calculated the experimental means and 95% CI for the extracellular concentration change rate (in mmol/[h·10^{12}RBC]) in the medium for 24 metabolites. Thus, we multiplied the

corresponding values for the relative change rates (as obtained above) and the initial concentrations (in mmol/l) of the metabolites in the medium [3] and divided the products by the RBC concentration of 0.11 trillion/l (10^{12} RBC/l) as calculated from a hematocrit level of 1% [2]. Although the lack of initial conditions for the other 27 metabolites prevented us from estimating their concentration change rates, for each of these metabolites, we qualitatively labeled the experimental concentration change direction as an increase (or a decrease) if the 95% CI of the metabolite's relative change rates included only positive (or negative) values. We were unable to determine the direction for a metabolite for which the corresponding 95% CI included both positive and negative values.

Step II: *Determining the computational exchange rates*

Given that the metabolites' extracellular concentration changes stemmed from their uptake or secretion between RBCs and the medium, we calculated the computational exchange rates by *1*) minimizing the differences between the computational rates and experimental means of the concentration changes for metabolites with available experimental means (the aforementioned 24 metabolites) and *2*) minimizing the discrepancy in directions between the computational exchanges and experimental concentration changes for metabolites with available experimental observations (part of the 27 metabolites mentioned above). We did this by solving the following optimization problem:

$$\min \sum_{j \in RE, RI, RD} L_j$$

s.t. $\left| v_j - v_{\exp,j} \right| \le L_j$ for exchange reaction *j* in *RE*

$v_j + L_j \ge 0$ for exchange reaction *j* in *RI*

$v_j - L_j \le 0$ for exchange reaction *j* in *RD*

$$L_j \geq 0$$

$$\mathbf{S}_{RBC} \cdot \mathbf{v} = \mathbf{0}$$

$$\mathbf{lb} \leq \mathbf{v} \leq \mathbf{ub}$$

$$\mathbf{S}' \cdot \mathbf{v} = \mathbf{0}$$

where L_j comprises non-negative slack variables, RE denotes the set of the exchange reactions of the 24 metabolites for which we estimated the experimental means of concentration change rates in *step I*, $v_{\exp,j}$ indicates the estimated mean rate for the metabolite with exchange reaction j and the corresponding v_j denotes the computational exchange rate for which positive and negative values indicate secretion and uptake of the metabolite, RI and RD represent the set of exchange reactions of metabolites for which we only determined the experimental concentration change directions as increases and decreases, respectively, $\mathbf{S}_{RBC}$, **lb,** and **ub** indicate the stoichiometric matrix, lower bounds, and upper bounds, respectively, of the fluxes derived from the RBC metabolic network [1], and **S'** comprises the coefficients for additional constraints, which set the flux through the pentose phosphate pathway and that through the Rapoport-Luebering shunt to be 3.4% and 145%, respectively, of the glucose uptake rate [4].

Step III: *Minimizing uptake rates and overall fluxes*

Due to the non-unique nature for the solution of the optimization problem in *step II*, we selected the metabolic flux distribution satisfying minimal uptake rates and minimal overall fluxes by sequentially solving two optimization problems. The first problem was as follows:

$$\min \sum_{j \in RU} \frac{v_j}{\alpha_j}$$

$$\text{s.t.} \quad \left| v_j - v_{\exp,j} \right| \leq L_j^* \qquad \text{for exchange reaction } j \text{ in } RE$$

$v_j + L^*_j \geq 0$ for exchange reaction j in *RI*

$v_j - L^*_j \leq 0$ for exchange reaction j in *RD*

$$\mathbf{S}_{RBC} \cdot \mathbf{v} = \mathbf{0}$$

$$\mathbf{lb} \leq \mathbf{v} \leq \mathbf{ub}$$

$$\mathbf{S}' \cdot \mathbf{v} = \mathbf{0}$$

where *RU* represents the set of uptake reactions of the metabolites for which we determined neither the rates nor directions of the experimental concentration changes and L_j^* was derived from the solution of the optimization in *step II*. α_j comprises the concentrations in Roswell Park Memorial Institute (RPMI) 1640 medium [3] of the metabolites for uptake reaction j in *RU*. If the concentration for a metabolite was unavailable, we arbitrarily set it to a small value (10^{-6} mmol/l).

Given the solution of the above equation, we finally calculated the fluxes of uninfected RBCs by minimizing the overall fluxes, as follows:

$$\min \sum_j |v_j|$$

s.t. $|v_j - v_{\exp,j}| \leq L^*_j$ for exchange reaction j in *RE*

$v_j + L^*_j \geq 0$ for exchange reaction j in *RI*

$v_j - L^*_j \leq 0$ for exchange reaction j in *RD*

$$\mathbf{S}_{RBC} \cdot \mathbf{v} = \mathbf{0}$$

$$\mathbf{lb} \leq \mathbf{v} \leq \mathbf{ub}$$

$$\mathbf{S}' \cdot \mathbf{v} = \mathbf{0}$$

$v_j \leq v^*_j$ for uptake reaction j in *RU*

where v_j^* comprises the fluxes through uptake reaction j in RU in the solution of the first optimization problem in *step III*.

RESULTS

First, we attempted to use the original *i*AB-RBC-283 metabolic network of RBCs [1] to reproduce the experimental concentration changes of the metabolites in the medium. We calculated the metabolic fluxes of uninfected RBCs, including the secretion or uptake fluxes of the metabolites present in the medium, and compared these computational exchange fluxes with the experimental concentration change rates for 24 metabolites for which the experimental rates were derived from metabolomic data [2] and initial concentration [3]. Table 1 shows that the computational rates were 0 for 18 metabolites, for 11 of which, such as arginine, the rates were within the experimental 95% CI, indicating quantitative agreements between computational results and experimental data for these metabolites. However, the disagreement of the remaining seven metabolites indicated that the RBC network was unable to capture the decrease in the concentration of *R*-pantothenate and the increases in thiamin, methionine, phenylalanine, (iso)leucine, tryptophan, and valine.

To address the above discrepancies, we further modified the *i*AB-RBC-283 metabolic network [1]. Due to the lack of the *R*-pantothenate consumption pathway in *i*AB-RBC-283, we added the pantothenate kinase reaction, which converts *R*-pantothenate into coenzyme A, in the global human metabolic network Recon1 [5]. Given the inability of human to synthesize thiamin (vitamin B_1), we added a hypothetical reaction of thiamin accumulation, by assuming that this metabolite's concentration increase in the medium was due to the secretion of molecules already contained in RBCs before the initial time point. We also added a degradation reaction of Albumax II (lipid-rich bovine serum albumin protein) and set the related coefficients of amino

acids and lipids based on the protein's amino acid sequence [6] and experimentally measured lipid compositions [7], respectively. This was done based on the assumption that methionine, phenylalanine, (iso)leucine, tryptophan, and valine could be generated through the degradation of Albumax II protein in the medium [2] by proteolytic enzymes of RBCs [8, 9], given the lack of *de novo* syntheses of these amino acids in humans.

Finally, given the modified metabolic network, we recalculated the metabolic fluxes and compared the computational and experimental changes in metabolite concentrations in the medium. Table 2 shows the quantitative agreements for most metabolites (20 of 24 metabolites). For the other four metabolites, their computational and experimental concentration changes did not contradict each other, i.e., an experimental concentration increase was matched by a computational decrease, or vice versa.

The change in the number of agreements/disagreements (from 17/7 to 20/4 in the improved model) and the decrease in the root mean square error in the measured metabolite rated (from 0.045 to 0.030 in the improved model) show the improvement after the implemented modification. Therefore, we used the modified *i*AB-RBC-283 network and the recalculated fluxes (v_j^{nRBC}) of uninfected normal RBCs for further calculation, as described in the main text.

Table 1. Comparison of experimental extracellular metabolite concentration change rates and computational values based on the original *i*AB-RBC-283 metabolic network

Rates are expressed as millimoles per hour per 10^{12} red blood cells (mmol/[h·10^{12}RBC]). The positive and negative values of the rates indicate increases and decreases in the concentrations of the corresponding metabolites in the medium, respectively. For each metabolite, we assessed the agreement between the results based on whether the computational rate was within the corresponding experimental 95% confidence interval (CI) [2].

Metabolite	Experimental rate		Computational rate	Agreement
	Mean	95% CI		
Arginine	2.2×10^{-3}	$[-7.1, 7.5]\times10^{-2}$	0	Yes
Choline	1.8×10^{-3}	$[-0.1, 3.6]\times10^{-3}$	0	Yes
Glucose	-5.9×10^{-1}	$[-1.3, 0.1]\times10^{0}$	-5.9×10^{-1}	Yes
Glutamine	2.4×10^{-1}	$[-1.2, 6.0]\times10^{-1}$	2.4×10^{-1}	Yes
Hypoxanthine	-3.5×10^{-3}	$[-9.9, 3.0]\times10^{-3}$	-3.5×10^{-3}	Yes
Methionine	3.5×10^{-2}	$[1.2, 5.7]\times10^{-2}$	0	No
Nicotinamide	-6.5×10^{-5}	$[-8.0, 6.5]\times10^{-4}$	0	Yes
Phenylalanine	4.5×10^{-2}	$[1.7, 7.3]\times10^{-2}$	0	No
Pyridoxine	-5.1×10^{-4}	$[-8.2, -2.1]\times10^{-4}$	-5.1×10^{-4}	Yes
Riboflavin	8.3×10^{-5}	$[-0.1, 16.4]\times10^{-5}$	0	Yes
Thiamin	4.1×10^{-4}	$[0.3, 7.8]\times10^{-4}$	0	No
(Iso)Leucine	2.0×10^{-1}	$[0.3, 3.7]\times10^{-1}$	0	No
Asparagine	1.4×10^{-2}	$[-3.2, 6.1]\times10^{-2}$	0	Yes
Aspartate	-2.0×10^{-3}	$[-1.2, 0.8]\times10^{-2}$	0	Yes
Glutamate	1.2×10^{-2}	$[-0.3, 2.6]\times10^{-2}$	1.2×10^{-2}	Yes
Histidine	2.4×10^{-3}	$[-1.2, 1.7]\times10^{-2}$	0	Yes
Inositol	-1.4×10^{-2}	$[-2.6, -0.1]\times10^{-2}$	-1.4×10^{-2}	Yes
4-aminobenzoate	1.8×10^{-4}	$[-0.6, 1.0]\times10^{-3}$	0	Yes
R-pantothenate	-4.4×10^{-5}	$[-6.9, -1.8]\times10^{-5}$	0	No
Proline	6.2×10^{-4}	$[-2.5, 2.6]\times10^{-2}$	0	Yes
Serine	-9.3×10^{-3}	$[-4.8, 3.0]\times10^{-2}$	0	Yes
Tryptophan	1.4×10^{-2}	$[0.6, 2.3]\times10^{-2}$	0	No
Tyrosine	1.5×10^{-2}	$[-0.3, 3.2]\times10^{-2}$	0	Yes
Valine	6.1×10^{-2}	$[2.8, 9.4]\times10^{-2}$	0	No

Table 2. Comparison of experimental extracellular metabolite concentration change rates and computational values based on the modified *i*AB-RBC-283 metabolic network

Rates are expressed as mmol/(h·10^{12} RBC). The positive and negative values of the rates indicate increases and decreases in the concentrations of the corresponding metabolites in the medium, respectively. For each metabolite, we assessed the agreement between the results based on whether the computational rate was within the corresponding experimental 95% confidence interval (CI) [2].

Metabolite	Experimental rate		Computational rate	Agreement
	Mean	95% CI		
Arginine	$2.2×10^{-3}$	$[-7.1, 7.5]×10^{-2}$	$2.2×10^{-3}$	Yes
Choline	$1.8×10^{-3}$	$[-0.1, 3.6]×10^{-3}$	$7.6×10^{-4}$	Yes
Glucose	$-5.9×10^{-1}$	$[-1.3, 0.1]×10^{0}$	$-5.9×10^{-1}$	Yes
Glutamine	$2.4×10^{-1}$	$[-1.2, 6.0]×10^{-1}$	$2.4×10^{-1}$	Yes
Hypoxanthine	$-3.5×10^{-3}$	$[-9.9, 3.0]×10^{-3}$	$-3.4×10^{-3}$	Yes
Methionine	$3.5×10^{-2}$	$[1.2, 5.7]×10^{-2}$	$4.0×10^{-3}$	No
Nicotinamide	$-6.5×10^{-5}$	$[-8.0, 6.5]×10^{-4}$	0	Yes
Phenylalanine	$4.5×10^{-2}$	$[1.7, 7.3]×10^{-2}$	$2.7×10^{-2}$	Yes
Pyridoxine	$-5.1×10^{-4}$	$[-8.2, -2.1]×10^{-4}$	$-5.1×10^{-4}$	Yes
Riboflavin	$8.3×10^{-5}$	$[-0.1, 16.4]×10^{-5}$	0	Yes
Thiamin	$4.1×10^{-4}$	$[0.3, 7.8]×10^{-4}$	$4.1×10^{-4}$	Yes
(Iso)Leucine	$2.0×10^{-1}$	$[0.3, 3.7]×10^{-1}$	$7.5×10^{-2}$	Yes
Asparagine	$1.4×10^{-2}$	$[-3.2, 6.1]×10^{-2}$	$1.4×10^{-2}$	Yes
Aspartate	$-2.0×10^{-3}$	$[-1.2, 0.8]×10^{-2}$	$4.0×10^{-2}$	No
Glutamate	$1.2×10^{-2}$	$[-0.3, 2.6]×10^{-2}$	$1.2×10^{-2}$	Yes
Histidine	$2.4×10^{-3}$	$[-1.2, 1.7]×10^{-2}$	$1.7×10^{-2}$	Yes
Inositol	$-1.4×10^{-2}$	$[-2.6, -0.1]×10^{-2}$	$-1.4×10^{-2}$	Yes
4-Aminobenzoate	$1.8×10^{-4}$	$[-0.6, 1.0]×10^{-3}$	0	Yes
R-Pantothenate	$-4.4×10^{-5}$	$[-6.9, -1.8]×10^{-5}$	$-4.4×10^{-5}$	Yes
Proline	$6.2×10^{-4}$	$[-2.5, 2.6]×10^{-2}$	$2.8×10^{-2}$	No
Serine	$-9.3×10^{-3}$	$[-4.8, 3.0]×10^{-2}$	$2.8×10^{-2}$	Yes
Tryptophan	$1.4×10^{-2}$	$[0.6, 2.3]×10^{-2}$	$2.0×10^{-3}$	No
Tyrosine	$1.5×10^{-2}$	$[-0.3, 3.2]×10^{-2}$	$2.0×10^{-2}$	Yes
Valine	$6.1×10^{-2}$	$[2.8, 9.4]×10^{-2}$	$3.6×10^{-2}$	Yes

Text S2. Model sensitivity to high-frequency gene expression variations

The modeling framework captured *Plasmodium falciparum* metabolic fluxes across 1,024 unique reactions and 924 metabolite species contained in three compartments as driven by a time-dependent gene expression profiles across the intraerythrocytic development cycle (IDC). This system was constrained by stoichiometry and dependent on the gene expression levels to generate feasible computational solutions to the optimization problems defined by the model.

Technical and biological factors associated with the gene expression data can contribute to potential model errors, such as experimental variations in the measured data and uncertainties in the association between transcript variations and enzyme variations. These uncertainties and variations influence the constrained solutions and may lead to an anomalous redistribution of fluxes if the expression levels have high variability between measured time points, i.e., the model becomes too sensitive to the expression variations. Thus, while the observed overall time-dependent gene-expression levels drive the IDC, the model constraints coupled with high variability expression data may force an artificial redistribution of fluxes that is manifested as high-frequency noise in hourly spikes and non-smooth variations in biomass production levels during the IDC.

Thus, instead of deriving the biomass production levels from only one simulation based on the given expression data, we created an ensemble of simulations using the experimental expression levels as a baseline, but with a small amount of added noise, and averaged the calculated results. We added normally distributed random noise of zero mean and 10% standard deviation to the expression values of a gene g at time t, i.e., $\hat{g}_t = g_t + f(\mu,\sigma)$, where $f(\mu,\sigma)$ is a normal probability distribution with mean $\mu = 0$ and standard deviation $\sigma = 0.1 \cdot g_t$, g_t is noise-free gene expression data, and $\hat{g}_t$ is the gene expression data after the noise addition. We repeated this procedure 20

times, calculated the metabolic fluxes during the IDC for each instance, and calculated a final averaged flux results for each metabolite. To ensure reproducibility of the results, we generated all random numbers using the same random generator algorithm [10] and recorded the seeds.

Additional file 1: Figure S2 (page 13 below) shows the results for the biomass production levels using this procedure. The effect is to minimize the hourly spikes, providing an overall smoothing that diminishes the exaggerated sensitivity of the model to gene expression variaions. This is noticeable in the reduction in variations associated with highly variable DNA and ubiquinol-8 production levels during the trophozoite stage and an overall smoothing out of the co-factor production levels. Thus, we can attribute the bulk of the observed high-frequency variability seen in these metabolite levels to an artificial high sensitive of the model to gene expression variations. The overall observations based on the averaged data discussed in the manuscript of general, low-frequency stage-specific trends and their differences are robust with respect to this model behavior.

Figure S1. Macromolecule syntheses in *Plasmodium falciparum* HB3 during the intraerythrocytic developmental cycle. The synthesized amount of RNA (A), protein (B), DNA (C), and phospholipids (D) during a given time interval was equal to the amount of material synthesized during the interval normalized by the maximum amount produced during the entire cycle. The predicted amounts were compared with the corresponding experimental data for DNA and RNA [11] and for phospholipids [12]. The predictions of the new modeling framework closely correspond to our earlier developed model that did not explicitly treat host red blood cell metabolism [13], however, the results are not quantitatively the same. The horizontal bars indicate the length of the time intervals. The colors of these bars represent the simulation results (blue) and experimental data (green).

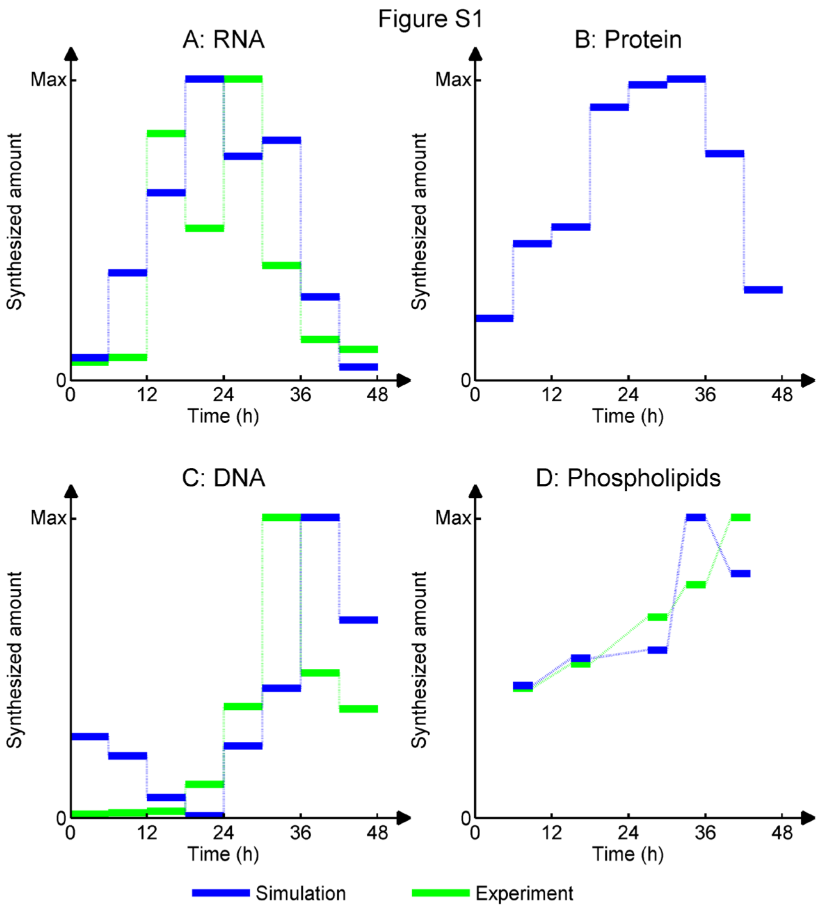

Figure S2. Predicted time-dependent production of biomass metabolites for the HB3, 3D7, and Dd2 strains of *Plasmodium falciparum* using 10% expression level variation. The average predicted time-dependent production levels of each biomass metabolite of *P. falciparum* during the intraerythrocytic developmental cycle as calculated from 20 simulations using a 10% Gaussian noise in gene expression levels. Error bars represent standard deviation of the calculated mean.

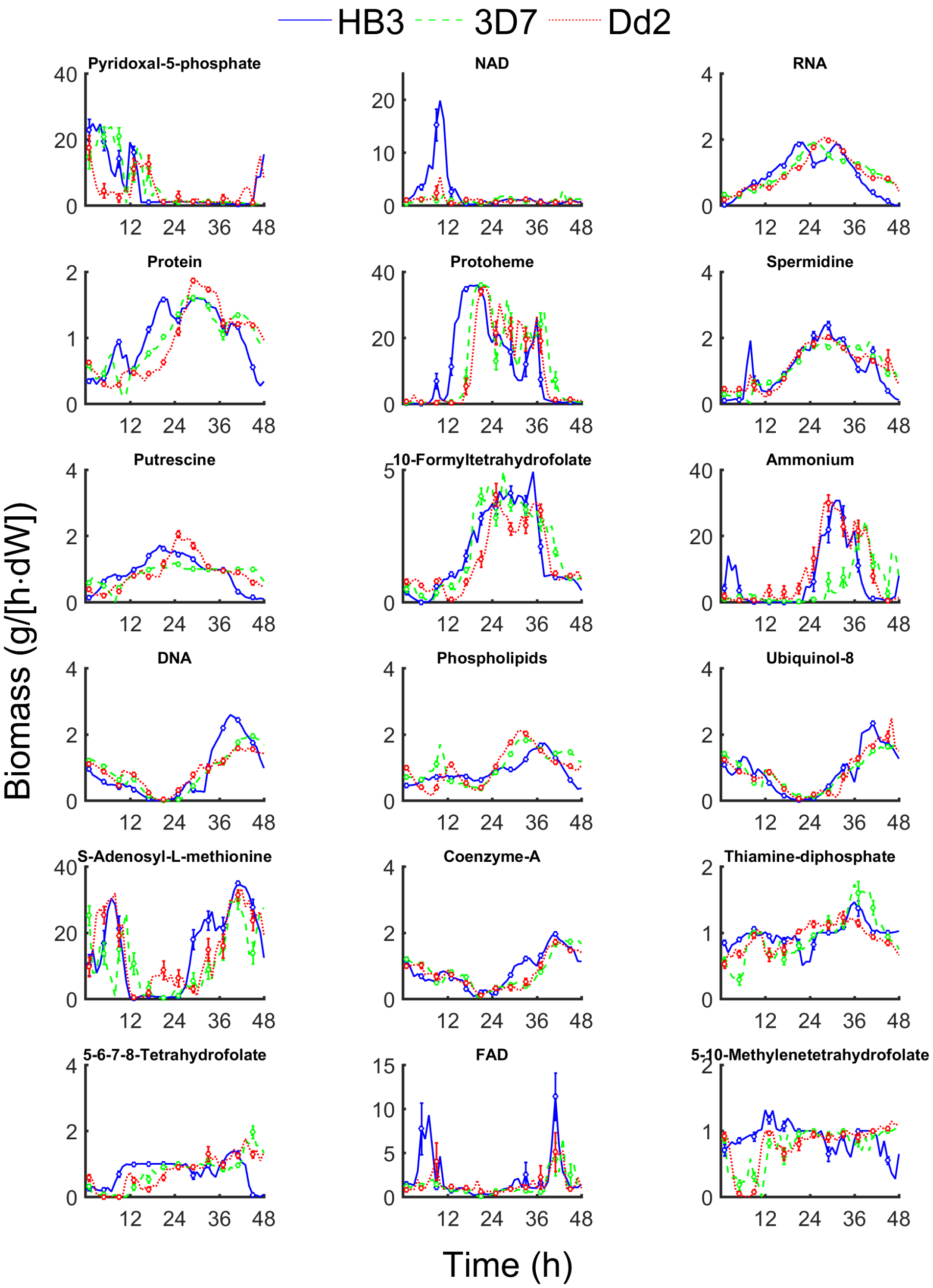

Figure S3. Predicted metabolic fluxes through the tricarboxylic acid cycle in three strains of *Plasmodium falciparum*. *Left*: predicted time-dependent flux profiles of the reactions in the tricarboxylic acid cycle of the HB3, 3D7, and Dd2 strains of *P. falciparum*. *Right*: rescaled flux profile of aconitate hydratase (ACONT), citrate synthase (CS), and isocitrate dehydrogenase (ICDH) within these strains. Fluxes are expressed as mmol/(h·10^{12} RBC). AKGDH, α-ketoglutarate dehydrogenase; FUM, fumarase; MDH, L-malate dehydrogenase; SUCD, succinate dehydrogenase; SUCOAS, succinate-coenzyme A ligase

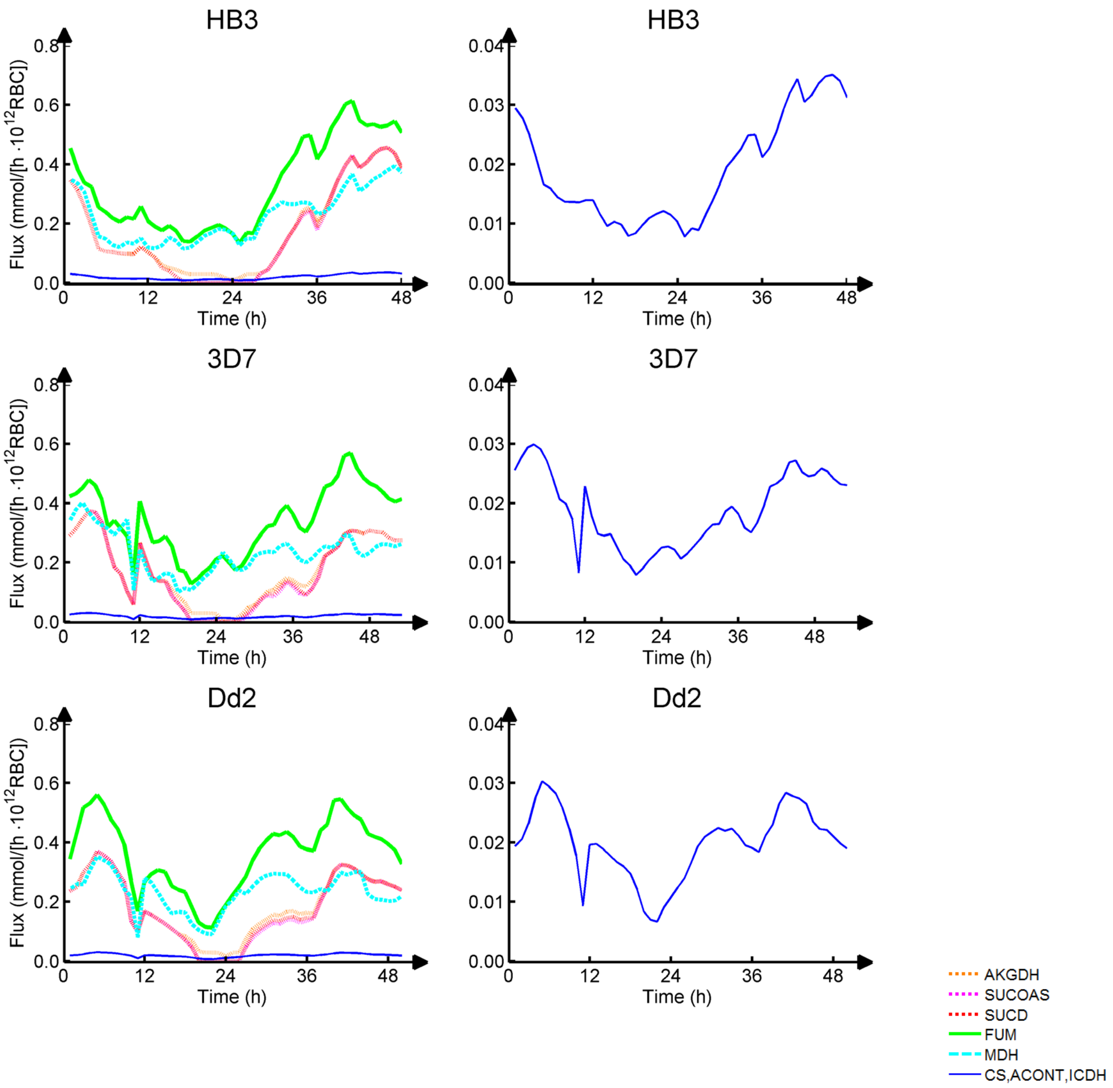

.

Figure S4. Flux ratios for the reactions in the glycolysis pathways of human red blood cells infected with *Plasmodium falciparum* 3D7 and Dd2 strains. The time-dependent ratios were defined as the ratios of reaction fluxes in red blood cells (RBCs) infected with *P. falciparum* HB3 (A) or Dd2 (B), to those in normal RBCs in the uninfected RBC culture. The time-dependent ATP transports were the transports from the related *P. falciparum* strains to the host RBCs. ATP transport flux is expressed as mmol/(h·10^{12} RBC). DPGase, diphosphoglycerate phosphatase; DPGM, diphosphoglycero mutase; ENO, enolase; FBA, fructose bisphosphate aldolase; GAPD, glyceraldehyde-3-phosphate dehydrogenase; HEX, hexokinase; iRBC, RBCs infected with *P. falciparum*; LDH, lactate dehydrogenase; nRBC, normal RBCs; PFK, phosphofructokinase; PGI, glucose-6-phosphate isomerase; PGK, phosphoglycerate kinase; PGM, phosphoglycerate mutase; PYK, pyruvate kinase; TPI, triose-phosphate isomerase.

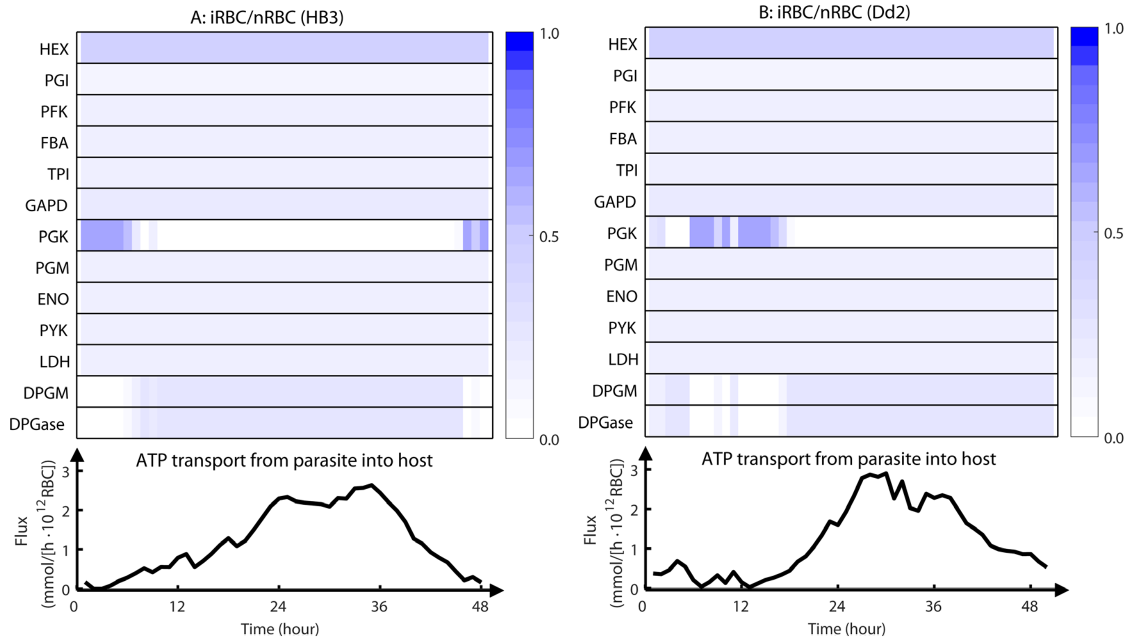

References

1. Bordbar, A., N. Jamshidi, and B.O. Palsson, *iAB-RBC-283: A proteomically derived knowledge-base of erythrocyte metabolism that can be used to simulate its physiological and patho-physiological states.* BMC Syst Biol, 2011. **5**: p. 110.
2. Olszewski, K.L., et al., *Host-parasite interactions revealed by Plasmodium falciparum metabolomics.* Cell Host Microbe, 2009. **5**(2): p. 191-9.
3. Moore, G.E. and L.K. Woods, *Culture media for human cells—RPMI 1603, RPMI 1634, RPMI 1640 and GEM 1717.* TCA manual / Tissue Culture Association, 1977. **3**(1): p. 503-509.
4. Lewis, I.A., et al., *Role of band 3 in regulating metabolic flux of red blood cells.* Proc Natl Acad Sci U S A, 2009. **106**(44): p. 18515-20.
5. Duarte, N.C., et al., *Global reconstruction of the human metabolic network based on genomic and bibliomic data.* Proc Natl Acad Sci U S A, 2007. **104**(6): p. 1777-82.
6. *National Center for Biotechnology Information*. Available from: http://www.ncbi.nlm.nih.gov/.
7. Garcia-Gonzalo, F.R. and J.C. Izpisua Belmonte, *Albumin-associated lipids regulate human embryonic stem cell self-renewal.* PLoS One, 2008. **3**(1): p. e1384.
8. Pontremoli, S., et al., *Identification of proteolytic activities in the cytosolic compartment of mature human erythrocytes.* Eur J Biochem, 1980. **110**(2): p. 421-30.
9. Sacchetta, P., et al., *Purification of human erythrocyte proteolytic enzyme responsible for degradation of oxidant-damaged hemoglobin. Evidence for identifying as a member of the multicatalytic proteinase family.* Biochim Biophys Acta, 1990. **1037**(3): p. 337-43.
10. Matsumoto, M. and T. Nishimura, *Mersenne twister: a 623-dimensionally equidistributed uniform pseudo-random number generator.* ACM Transactions on Modeling and Computer Simulation (TOMACS), 1998. **8**(1): p. 3-30.
11. Gritzmacher, C.A. and R.T. Reese, *Protein and nucleic acid synthesis during synchronized growth of Plasmodium falciparum.* J Bacteriol, 1984. **160**(3): p. 1165-7.
12. Vial, H.J., M.J. Thuet, and J.R. Philippot, *Phospholipid biosynthesis in synchronous Plasmodium falciparum cultures.* J Protozool, 1982. **29**(2): p. 258-63.
13. Fang, X., J. Reifman, and A. Wallqvist, *Modeling metabolism and stage-specific growth of Plasmodium falciparum HB3 during the intraerythrocytic developmental cycle.* Mol Biosyst, 2014. **10**(10): p. 2526-37.
